# Myosin VI is involved in the structural organization and molecular composition of epididymal epithelial cells in mouse^†^

**DOI:** 10.1093/biolre/ioag031

**Published:** 2026-02-02

**Authors:** Anna Richert, Robert Lenartowski, Piotr Wasąg, Przemysław Zakrzewski, Joanna Suszyńska-Zajczyk, Olena Karatsai-Miaskowska, Maria Jolanta Rędowicz, Marta Lenartowska

**Affiliations:** Department of Cellular and Molecular Biology, Faculty of Biological and Veterinary Sciences, Nicolaus Copernicus University in Toruń, Torun, Poland; Department of Cellular and Molecular Biology, Faculty of Biological and Veterinary Sciences, Nicolaus Copernicus University in Toruń, Torun, Poland; Centre for Modern Interdisciplinary Technologies, Nicolaus Copernicus University in Toruń, Torun, Poland; Department of Cellular and Molecular Biology, Faculty of Biological and Veterinary Sciences, Nicolaus Copernicus University in Toruń, Torun, Poland; School of Cellular and Molecular Medicine, Faculty of Health and Life Sciences, University of Bristol, Bristol, United Kingdom; Department of Biochemistry and Biotechnology, Faculty of Agriculture, Horticulture and Biotechnology, Poznań University of Life Sciences, Poznan, Poland; Laboratory of Molecular Basis of Cell Motility, Nencki Institute of Experimental Biology, Polish Academy of Sciences, Warsaw, Poland; Laboratory of Molecular Basis of Cell Motility, Nencki Institute of Experimental Biology, Polish Academy of Sciences, Warsaw, Poland; Department of Cellular and Molecular Biology, Faculty of Biological and Veterinary Sciences, Nicolaus Copernicus University in Toruń, Torun, Poland; Centre for Modern Interdisciplinary Technologies, Nicolaus Copernicus University in Toruń, Torun, Poland

**Keywords:** actin cytoskeleton, APPL1, clathrin, Dab2, endocytosis, epididymal epithelium, GIPC1, Myosin vi, Snell’s waltzer mice

## Abstract

Myosin VI (MYO6) is the only actin-based motor protein that moves toward the minus end of actin filaments. It participates in multiple cellular processes, including endocytosis, secretion, autophagy, and the formation of apical stereocilia and microvilli in highly specialized epithelia. These diverse functions are mediated by specific cargo-adaptor proteins that recruit MYO6 to distinct cellular compartments. We have previously demonstrated that loss of MYO6 function in Snell’s waltzer mice leads to several defects during spermatogenesis, resulting in reduced male fertility. Here, we show for the first time that MYO6 and selected binding partners are differentially expressed in mouse epididymal epithelium, a highly specialized mammalian epithelia developing apical microvilli. Using immunocytochemistry, confocal microscopy, and biochemical approaches we found that: (i) MYO6 is present in the epithelium of the common efferent duct and all segments of the epididymis, (ii) MYO6 and Dab2 colocalize predominantly at the apical surface of epithelial cells in the efferent duct and caput, (iii) MYO6 and GIPC1 are mainly detected in epithelial cells in the caput and corpus, with the lowest level observed in the cauda. Moreover, depletion of MYO6 results in altered distribution of clathrin and APPL1 in epididymal epithelial cells and causes ultrastructural abnormalities. Altogether, our findings indicate that MYO6 contributes to the endocytic pathway in the mouse epididymal epithelium, a process essential for generating the microenvironment required for sperm maturation. In addition, MYO6 supports the structural organization of apical microvilli, thereby facilitating sperm transport through the epididymal duct.

## Teaser

Myosin VI, a unique actin-based motor protein, is involved in the formation and function of the mouse epididymal epithelium through interaction with specific cargo adapter proteins.

## Graphical Abstract

**Graphical Abstract:**
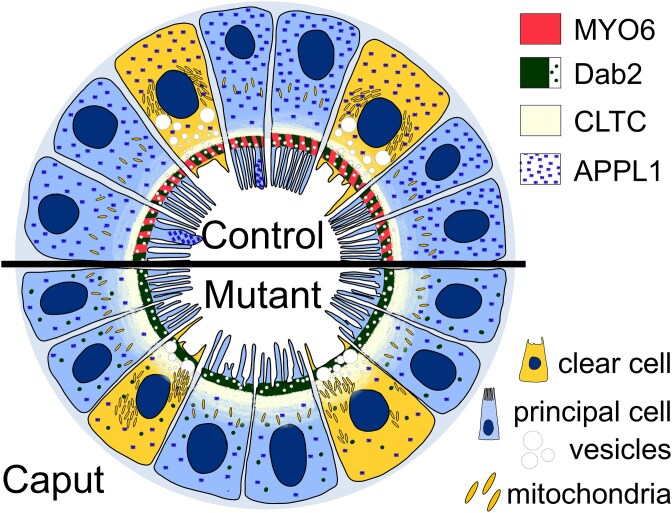

## Introduction

Unconventional myosin of class VI (MYO6) is a molecular motor that moves towards the minus end of actin filaments, in the opposite direction to other myosins characterized to date [1]. This unique feature may explain involvement of MYO6 in a range of specific cellular pathways in animal cells, such as endocytosis, receptor trafficking, protein secretion, autophagy, and regulation of actin organization and dynamics, including formation and maintenance of apical microvilli and stereocilia in polarized epithelial cells [2–5]. In these processes, MYO6 may function as a motor involved in intracellular transport or as an anchor essential for tethering membranes or protein complexes to the actin cytoskeleton. Importantly, loss of *Myo6* expression, as observed in knock out (KO) Snell’s waltzer mice (*sv*/*sv*) or in humans with mutations in the *MYO6* gene, causes several diseases, including deafness [6], astrogliosis [7], proteinuria [8], and hypertrophic cardiomyopathy [9]. Overall, the structure of MYO6 heavy chain is typical of myosin superfamily and includes an N-terminal motor domain (head) that contains an ATP-binding pocket and directly interacts with microfilaments, a “lever arm” (neck) that binds calmodulin/calmodulin-like chains, and a tail that contains C-terminal cargo binding domain [2]. MYO6 also has two unique inserts, a class-specific insert-2 (53-aa, located between the converter and the calmodulin-binding IQ motif) responsible for its “backward” movement [10] and insert-1 (26-aa, located in the motor domain) responsible for the rate of ATP-binding [11]. The multifunctionality of MYO6 requires interaction with diverse partners via its cargo binding domain which contains several unique regions, such as two adaptor-binding motifs RRL and WWY [2]. Moreover, the cargo-binding distal tail of MYO6 undergoes alternative splicing resulting in four isoforms containing the large insert (LI), small insert (SI), both inserts (L + S), or no insert (NI). Interactions with the specific adaptor proteins appear to be crucial for motor activation and the transition between monomeric and oligomeric states of MYO6 [3, 12]. On the other hand, the presence or absence of specific binding motifs and splice inserts determines the ability of MYO6 to interact with various cargo-adaptor proteins that link MYO6 to different cellular compartments and processes, including endocytic and exocytic pathways [3, 4]. It is generally accepted that MYO6-LI isoform is expressed predominantly in polarized cells at their apical plasma membrane and participates in clathrin-mediated endocytosis, whereas the MYO6-NI splice variant, which is widely expressed, associates mainly with uncoated endocytic vesicles. In the early endocytic pathway, recruitment of MYO6 to clathrin-coated pits and vesicles requires interaction with the clathrin adaptor Dab2 (Disabled-2) via the WWY motif [13]. It was also demonstrated that MYO6 targeting to clathrin-coated structures involves the MYO6-LI splice variant, which constitutes the clathrin-binding domain [14]. In contrast, through interaction with GIPC (GAIP interacting protein C-terminus) via RRL motif, MYO6 is recruited to Rab5- and APPL1-positive early endosomes and facilitates their movement through the actin-rich cell cortex away from the plasma membrane [15, 16]. In addition to the important role of MYO6 in endocytosis, this protein appears to be involved in the secretory pathway. In fibroblast cell lines, MYO6 is associated with both the Golgi apparatus and the leading edge of the cell, and is present in a cytosolic pool [17]. Functional studies using *sv*/*sv* mouse fibroblasts have shown a significant reduction in the size of the Golgi complex, coupled with some defects in exocytosis [17, 18]. Moreover, the MYO6-SI splice variant tethers secretory granules to the actin-rich cortex in neurosecretory cells [19].

**Figure 1:**
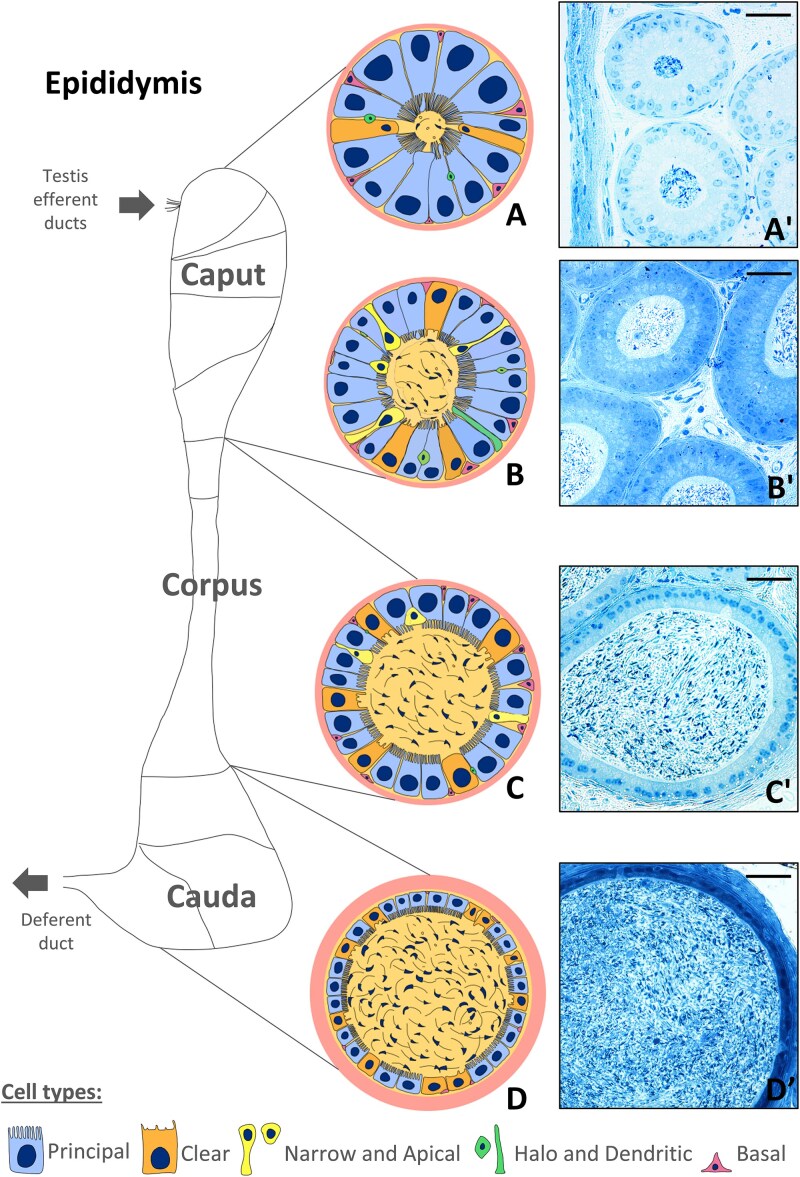
Overview of the mouse epididymis highlighting connections to the testis efferent ducts and anatomical segmentation of the epididymis. For each anatomical segment of the epididymis (initial segment, caput, corpus, cauda), a schematic diagram of the cross-section through the epididymal tubule (A–D) and the corresponding semi-thin sections stained with methylene blue (A’–D′) are shown. In the subsequent epididymal segments, differences in the height of epithelial cells (it decreases in more distal regions), the length of microvilli (it is shorter in successive regions), the number of sperm (it increases in more distal segments), and the variability of cellular composition are observed. The most abundant cells in the epididymal epithelium are the principal cells, which are tall, columnar cells with prominent apical microvilli. These cells display a high level of exo/endocytosis and participate in modification of the microenvironment in epididymal lumen to support sperm maturation. The secretory activity of principal cells (especially in the proximal segments of the epididymis) is reflected in the formation of exosome-like vesicles (epididysomes), which detach from the apical margin of epithelial cells to form large bleb-like structures. Principal cells may also participate in endocytosis, involving the uptake of epididymal luminal contents via receptor-mediated mechanism. Clear cells, which are particularly abundant in the distal epididymis, are characterized by cytoplasm rich in acidic vesicles and lysosomes. They are responsible for endocytosis and degradation of proteins from the luminal fluid, and contribute to lumen acidification, which is necessary to maintain sperm in a resting state. Narrow and apical cells are slender cells that play a role in proton secretion, supporting acidification of the epididymal lumen, which is essential for sperm maturation and storage. Another cell type, known as halo cells, represent immune cells (mainly lymphocytes), located at the base of the epithelium and associated with immune surveillance and local defense. Finally, small basal cells are located at the base of the epithelium and do not usually reach the lumen. Although basal cells do not directly involved in secretion, they are believed to act as sensors of the epithelial microenvironment and play a regulatory role in maintaining epithelial integrity and function [39]. Scale bar 50 μm.

MYO6 is required for the maintenance of brush border structures in highly specialized epithelia developing apical microvilli, such as intestinal and proximal tubule epithelia, as its deficiency in KO mice results in morphological and functional perturbations [8, 20–26]. MYO6 is also present in hair cells of the inner ear at the base of the stereocilia, and loss of its expression results in structural defects of stereocilia, impaired endocytosis, and deafness [6, 27–32], also in humans [33]. Recently, we found that the short isoforms of MYO6 are expressed in mouse testes and MYO6 depletion in KO mice causes defects during the acrosome biogenesis and leads to reduced male fertility [34–36]. We also demonstrated that MYO6, together with selected adaptor proteins, localizes to an early endocytic APPL1-positive compartment of highly specialized actin-rich structures, the apical tubulobulbar complexes [36]. These complexes mediate endocytosis of intercellular junctions between maturing spermatids and Sertoli cells, a crucial process for spermiation [37, 38]. Furthermore, we showed that the loss of MYO6 causes disorganization of the tubulobulbar complexes [36]. However, despite their essential role in sperm release, we did not observe major differences in sperm number in MYO6-deficient mice. This suggests that reduced male fertility observed in KO male mice may be due to additional factors. Sperm leaving the testis are immature, and their ability to fertilize an egg develops in the epididymis. The epididymis is a long and coiled tubule covered by a pseudostratified epithelium and divided into three main segments: caput (head), corpus (body), and cauda (tail) [39, 40, Figure 1]. Different epididymal epithelial cell types within these segments (principal, basal, narrow, apical, clear, halo, and dendritic cells) are responsible for creating a microenvironment that supports the sequential maturation of sperm (in the caput and corpus) and their subsequent storage (in the cauda). As shown in Figure 1, the initial segment (sub-region of the caput) and the caput are characterized by a narrow lumen diameter and low sperm concentration, while both lumen diameter and sperm concentration increase distally within the corpus and cauda of the epididymis. The predominant cells in the epididymal epithelium (~80%) are microvilli-developing principal cells, which are long in the caput and become progressively shorter toward the cauda [39]. The efferent ducts connecting the testis and epididymis occupy a significant portion of the caput in larger mammals and humans [41, 42], whereas in mice they comprise only a small, proximal region of the epididymis called the common efferent duct [41]. The tubules of the efferent duct are lined by a monolayer epithelium of ciliated and nonciliated cells and reabsorb up to 95% of the luminal fluids [43, 44]. Ciliated cells have motile cilia extending into the lumen between microvilli and provide a mechanism for moving sperm through the duct, whereas nonciliated cells contain an apical brush border of microvilli involved in absorptive function. The establishment of the unique microenvironment in the efferent duct and then in particular segments of the epididymis feature the varied endocytic and exocytic contributions of the epithelial cells [39, 44–47]. Given that MYO6 regulates these pathways in animal cells and that its deficiency is associated with reduced fertility in male mice, we decided to investigate the potential involvement of MYO6 in the functional organization of the mouse epididymal epithelium.

## Materials and methods

### Animals

Three-month-old male heterozygous control mice (*sv*/+) and Snell’s waltzer mutants (*sv*/*sv* C57BL/6 background) were used in the study. As a result of a spontaneous mutation of the *sv* allele in homozygous *sv*/*sv* mice, the presence of MYO6 was not detected in any of the tissues examined so far [23, 24], including testes [35, 36] and the epididymis (present work). The mutation is due to a 130 bp deletion, which causes the presence of a premature stop codon in the *Myo6* gene [28]. Unlike KO mice, *sv*/+ mice express MYO6 protein at levels similar to wild-type animals. Therefore, during functional studies pairs of control (*sv*/+) and mutant (*sv*/*sv*) male mice from the same litter were used. The animals were bred and housed under pathogen-free conditions at the Nencki Institute of Experimental Biology (Polish Academy of Sciences, Warsaw, Poland), and all animal procedures were carried out in accordance with the European Communities Council directives adopted by the Polish Parliament (Act of 15 January 2015 on the use of animals in scientific investigations). We have made every effort to minimize the suffering of the animals and used the smallest number of animals to obtain reliable results.

For preliminary studies, control and KO male mice, as well as dissected testes and epididymides, were weighed. The ratio of testes and epididymides mass to total body mass (T/B and E/B) was calculated using the following formula: T/B = testis mass (mg)/body mass (g); E/B = epididymis mass (mg)/body mass (g). Testes and epididymides were photographed using a Nikon D5300 camera equipped with a NIKON AF-S DX Nikkor 35 mm f/1.8G lens.

All experiments using different methods were performed in at least three independent biological replicates and representative data were presented.

### Co-immunoprecipitation and stain-free western blotting

Whole epididymides dissected from control and mutant mice were homogenized using a manual tissue grinder in ice-cold lysis buffer (50 mM Tris–HCl, pH 8.0; 100 mM NaCl; 1% Triton X-100; 5 mM ATP) supplemented with 1 × cOmplete™ Protease Inhibitor Cocktail (Roche). After 30 min of incubation on ice, lysates were centrifuged twice at 15.000 × g for 10 min at 4°C. Supernatants were collected, transferred to fresh tubes, and diluted 1:3 with immunoprecipitation (IP) buffer (50 mM HEPES, pH 7.5; 150 mM NaCl; 5 mM MgCl₂; 1% Triton X-100). For co-immunoprecipitation, lysates were incubated overnight at 4°C with end-over-end mixing in the presence of magnetic beads conjugated with 5 μg of either anti-Dab2 or anti-GIPC1 polyclonal antibodies (PAbs, Supplementary Table S1). After incubation, beads were sequentially washed with the following ice-cold buffers: Low Salt Wash Buffer (20 mM Tris–HCl, pH 8.0; 2 mM EDTA; 150 mM NaCl; 1% Triton X-100), High Salt Wash Buffer (20 mM Tris–HCl, pH 8.0; 2 mM EDTA; 500 mM NaCl; 1% Triton X-100), TE Buffer (10 mM Tris–HCl, pH 8.0; 1 mM EDTA). Bound proteins were eluted under denaturing conditions in loading buffer (LB) supplemented with dithiothreitol (DTT), run on 10% TGX gels (Bio-Rad), and semi-dry transferred onto Immun-Blot® Low Fluorescence PVDF Membrane (Bio-Rad). To visualize resolved proteins, stain-free gels were activated in ChemiDoc™ Touch Imaging System (Bio-Rad). Next, the blots were blocked with 0.5% (for Dab2 analysis) or 1% (for GIPC1 and MYO6) bovine serum albumin (BSA) in 1 × PBS-T buffer (phosphate-buffered saline with 0.1% Tween 20). Whole blots or parts thereof (blots were cut at the 75 kDa level to increase the signal of specific epitopes) and probed with the primary PAbs (Supplementary Table S1). After washing in 1 × PBS-T buffer, blots were probed with the anti-rabbit secondary IgG HRP antibody (Merck). The signal was detected using the Amersham ECL Advance Western Blotting Detection, according to the manufacturer’s protocol (GE Healthcare) and imaged in on ChemiDoc™ Touch Imaging System (Bio-Rad).

For verification of the specificity of the primary antibodies, immunoblotting was performed according to the protocol described previously [48], with some modifications. In brief, testes and epididymides dissected from *sv*/+ and *sv*/*sv* male mice were homogenized in liquid nitrogen. A soluble fraction of proteins was isolated using extraction buffer (50 mM Tris–HCl, pH 7.5; 0.5% Triton X-100; 150 mM NaCl; 5% glycerol) supplemented with 1 × cOmplete™ Protease Inhibitor Cocktail (Roche) according to the manufacturer’s recommendations. The homogenates were centrifuged at 16.000 × g for 20 min at 4°C, and protein concentration was measured using the DC Protein Assay (Bio-Rad) according to the manufacturer’s instructions. Equal amounts of protein (20 μg per well) were separated on 10% TGX gels (Bio-Rad) and fluorescently labeled with a trihalo compound using ChemiDoc™ Touch Imaging System (Bio-Rad). Next, the proteins were semi-dry transferred onto Immun-Blot® Low Fluorescence PVDF Membrane (Bio-Rad) and imaged again using ChemiDoc™ Touch Imaging System (Bio-Rad). The blocked blots were then probed with the primary PAbs (Supplementary Table S1) and visualized as described above.

### MYO6 splice variant analysis by RT-PCR

To identify MYO6 isoforms expressed in mouse epididymis, RT-PCR was performed as described by Zakrzewski et al. [34], with some modifications. Briefly, tissue samples (epididymis caput, corpus, and cauda) were homogenized in liquid nitrogen. Total RNA was isolated using the RNA Extracol (EURx) in accordance with the manufacturer’s guidelines. Subsequently, 2.5 μg of purified total RNA from each tissue sample was subjected to reverse transcription using dART reverse transcriptase and an oligo(dT)20 primer, following the manufacturer’s protocol (EURx). A 1 μL aliquot of the first-strand cDNA served as the template for the initial PCR in a nested PCR approach. The same volume of the first PCR product was subsequently used as the template for the second PCR to generate the final amplicons. Amplification was performed using SuperFi DNA polymerase (Thermo Fisher Scientific) with outer and inner gene-specific primers (Supplementary Table S2). PCR cycles were as follows: 95°C for 90 s, followed by 35 cycles of 95°C for 30 s, 60°C for 30 s, and 72°C for 30 s, followed by a final extension step of 72°C for 10 min. PCR products were resolved on a 2% agarose gel in 1× TAE buffer and visualized using the ChemiDoc™ Touch Imaging System (Bio-Rad).

### Immunocytochemistry and F-actin staining

For immunofluorescent localization of proteins in mouse epididymis, tissue samples (caput, corpus, and cauda) dissected from control and KO mice were fixed with 4% (v/v) formaldehyde (Polysciences) in 1 × PBS buffer (pH 7.4) overnight at 4°C. Fixed samples were washed with the same buffer and then saturated with increasing concentrations of sucrose (15%, 20%, 30% sucrose in 1 × PBS pH 7.4). Next, frozen tissue sections were sectioned using cryotome (Leica CM1520), collected on Superfrost™ Plus Adhesion Microscope Slides (Epredia), and warmed for 15 min at 37°C before immunolabeling. The sections were pre-incubated with 1% BSA and 0.1% saponin in 1 × PBS (pH 7.4) for 30 min at room temperature and labelled with the primary PAbs (Supplementary Table S1) in 1 × PBS supplemented with 0.1% BSA (overnight at 4°C). Next day, the sections were incubated with the anti-rabbit secondary IgG antibody Alexa Fluor Plus 594 (immunolabeling, Invitrogen) and phalloidin Alexa Fluor 488 (F-actin staining, Invitrogen) for 2 hours at room temperature, both diluted 1:100 in 1 × PBS with 0.15% BSA. Subsequently, DNA was stained using a Hoechst 33342 (Invitrogen) in mQ H_2_O (diluted 1:5000). The negative control was also performed by omitting the primary antibodies. Signal detection was performed using Olympus Fluoview FV 3000 confocal microscope. The acquired images were processed using the Olympus cellSens Dimension software (Build 18,987) and Fiji Image Processing Program [49].

### Histochemistry and transmission electron microscopy

For histological and ultrastructural analyses, samples of epididymis (caput, corpus, cauda) dissected from control and KO mice were fixed with 2.5% (v/v) glutaraldehyde (Sigma-Aldrich) in 0.2 M cacodylate buffer (pH 7.4) for 4 hours on ice. The samples were then fragmented into smaller pieces and further fixed in the same fixative solution overnight at 4°C. After washing, the samples were post-fixed with 2% (v/v) osmium tetroxide (Polysciences) in cacodylate buffer (pH 7.4) for 1 hour at 4°C and washed several times in the same buffer and mQ H_2_O. Finally, the tissue samples were dehydrated by passing them through the graded ethyl alcohol and embedded in Spurr resin (Sigma-Aldrich) following a standard protocol. The embedded specimens were sectioned using a diamond knife (Micro Star Technologies) and the Leica UTC ultramicrotome. Semi-thin sections (cross-sections of epididymal tubules) were transferred onto microscope slides, stained with 0.1% methylene blue, and analyzed using a Nikon Eclipse 80i microscope and NIS-Elements AR.3.00 software. Ultrathin cross-sections of epididymal tubules were sectioned using a diamond knife and the Leica UTC ultramicrotome, collected on copper grids, and stained with 2.5% uranyl acetate and 0.4% lead citrate solutions. Images were acquired using a Jeol 1010 transmission electron microscope at 80 kV equipped with a MegaView II camera.

### Statistical analysis

Each experiment was repeated in three biological replicates, performed for a pair of males (*sv*/+ and *sv*/*sv*) from the same litter, along with at least two technical replicates. The measurement of testis and epididymis weight, as well as their ratio to the total body weight of the male, was presented as the mean ± SD. For immunocytochemical studies, quantitative fluorescence measurements was also performed using representative tubules (set of serial optical sections) of individual epididymal epithelial segments, and Image J and GraphPad 8.0 software were used for statistical analyses and data visualization (graphs). For statistical analysis, a parametric unpaired *t*-test with Welch’s correction and a nonparametric unpaired Mann–Whitney test were performed. The data were considered statistically significant when *P* ≤ 0.05 (^*^), *P* ≤ 0.01 (^**^), *P* ≤ 0.001 (^***^), and *P* ≤ 0.0001 (^****^); exact p-values were summarized in Supplementary Tables S3–S4 and Supplementary Figures S2–S6.

## Results

### Macroscopic analysis and in vitro studies

**Figure 2:**
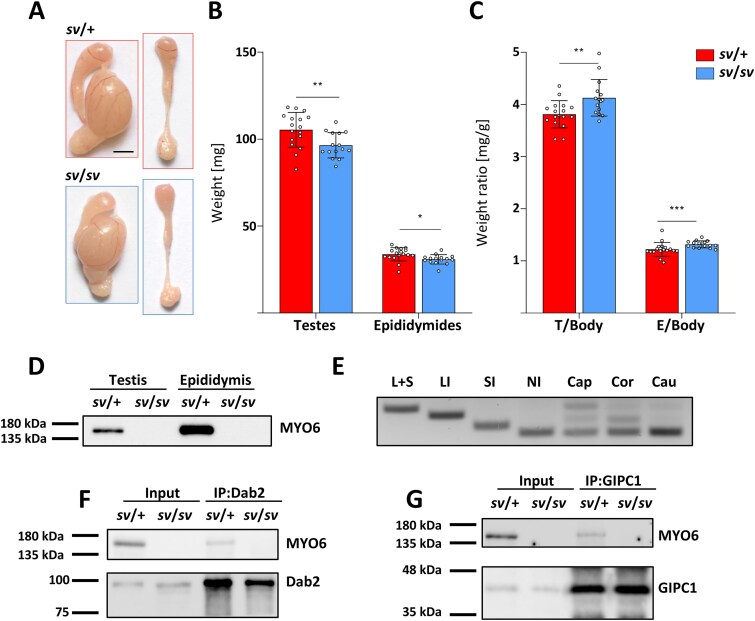
Morphological studies, immunoblotting, RT-PCR, and co-immunoprecipitation. Digital images (A) show mouse testes with attached epididymides and whole epididymides dissected from *sv*/+ control (*red frame*) and *sv*/*sv* mutant (*blue frame*) mice; scale bar 2.5 mm. The graphs present the analysis of the average weight of testes and epididymides (B) and the weight-to-body weight ratio (C) in control (*red*) and mutant (*blue*) mice. Error bars represent standard deviation, with statistical significance indicated as ^*^P ≤ 0.05, ^**^P ≤ 0.01, and ^***^P ≤ 0.001. Western blot analysis (D) of tissue extracts from *sv*/+ and *sv*/*sv* testes and epididymides were performed using an anti-MYO6 primary antibody. RT-PCR results (E) from control *sv*/+ male mice illustrate reference bands corresponding to MYO6 isoforms with both inserts (L + S), long insert (LI), short insert (SI), and without inserts (NI). Next lanes represent RNAs extracted from different regions of the epididymis: caput (*Cap*), corpus (*Cor*), and cauda (*Cau*). Co-immunoprecipitation (F, G) of MYO6, Dab2, and GIPC1 from *sv*/+ and *sv*/*sv* epididymis lysates. Dab2 (F) and GIPC1 (G) were immunoprecipitated with the primary antibodies against Dab2 and GIPC1. Total *sv*/+ homogenates (*input*) were used as positive controls and lysates of *sv/sv* were used in the negative control lane. The immunoprecipitates were analyzed by western blotting with the primary antibodies against MYO6, Dab2, and GIPC1.

To determine whether loss of MYO6 affects the male reproductive system, we first assessed the overall morphology of three-month-old mouse testes/epididymides dissected from control (*sv*/+) and Snell’s waltzer (*sv*/*sv*) mice (Figure 2A). The macroscopic analysis showed that organs dissected from *sv*/*sv* males were slightly smaller compared to the control organs. Indeed, the average weight of heterozygous gonads and epididymides was 105.26 mg (± 10.01 mg) and 33.75 mg (± 3.8 mg), respectively, [sample size (*n*) = 17], while in homozygous males, the weights were 96.46 mg (± 7.24 mg) and 30.86 mg (± 2.69 mg), respectively, [sample size (*n*) = 15] (Figure 2B). Thus, the weight of *sv*/*sv* testes was reduced by 8.44%, and the weight of *sv*/*sv* epididymides was reduced by 8.55% compared to the control organs. However, as shown in Figure 2C, testis/epididymis to body weight ratio of MYO6 mutant males was significantly larger than in control males (for T/B: *sv*/+ = 3.81 ± 0.26, *sv*/*sv* = 4.13 ± 0.35; for E/B: *sv*/+ = 1.22 ± 0.13, *sv*/*sv* = 1.32 ± 0.07), reflecting the difference in body weight between control and mutant males. It should be noted that Snell’s waltzer mice are smaller and they have less body fat compared to control mice [9, 50]. Western blot analysis confirmed complete loss of MYO6 expression in both testes and epididymis of *sv*/*sv* male mice (Figure 2D and Supplementary Figure S1A, A’).

In mammals, four MYO6 splice isoforms containing different inserts (LI or SI), both inserts (L + S), or no insert (NI) in the C-terminal globular tail can be expressed [3]. Because these specific isoforms are differentially expressed and have different functions in various tissues/cell types, we investigated which of them are expressed in defined segments of the mouse epididymis. To establish this, we performed RT-PCR and found that both shorter and longer MYO6 splice variants were detected in the caput, corpus, and cauda of control mice (Figure 2E and Supplementary Figure S1B). RT-PCR also showed that the MYO6-NI, LI and L + S isoforms are expressed in the caput and corpus, whereas the MYO6-NI isoform predominated in the cauda. We next determined which of the selected adaptor proteins could interact with MYO6 in the mouse epididymis. We focused mainly on representatives of these proteins that are known to interact with MYO6 isoforms with or without LI and are involved in different steps of clathrin-mediated endocytosis in polarized cells. Our co-immunoprecipitation experiments demonstrated that affinity-purified MYO6 PAb was able to pull down MYO6 together with Dab2 (Figure 2F and Supplementary Figure S1C) and GIPC1 (Figure 2G and Supplementary Figure S1D) from *sv*/+ epididymis. Remarkably, Dab2 and GIPC1 were detected both in immunoprecipitates and total lysates from *sv*/*sv* mice, indicating that their expression was not altered. No MYO6 was observed in lysates from *sv*/*sv* epididymis used as a negative control. These results indicate that in the mouse epididymis, MYO6 is present in a complex with its binding partners Dab2 and GIPC1, which link MYO6 to different stages of the endocytic pathway.

### Immunolocalization of MYO6 in mouse epididymal epithelium

**Figure 3:**
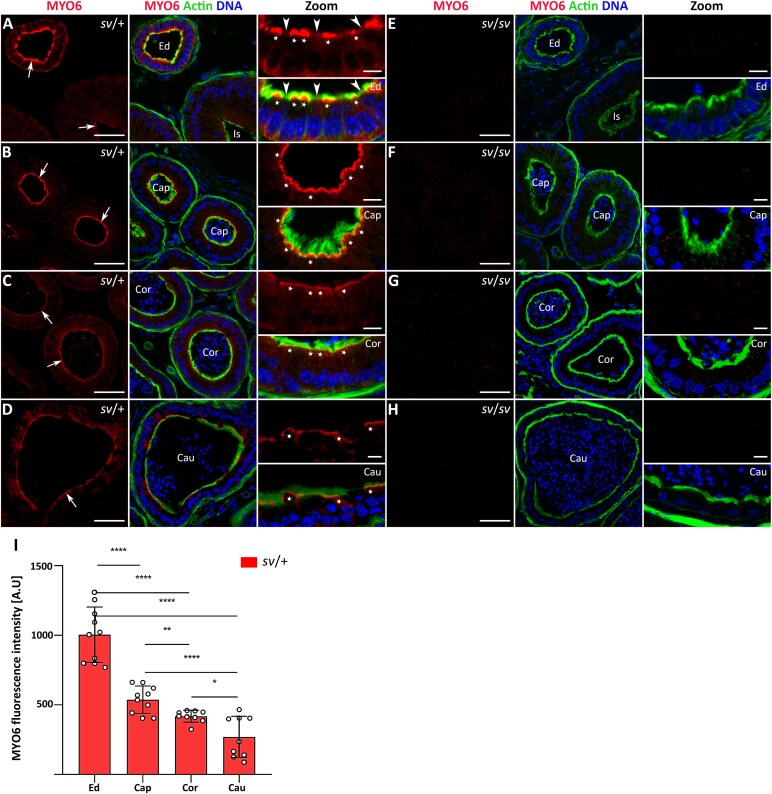
Immunolocalization of MYO6 in the epididymal epithelium of control mice (A–D) and Snell’s waltzer mutants (E–H), and statistical analysis of the fluorescence intensity (I). The following panels show: the common efferent duct and initial segment (A, E), caput (B, F), corpus (C, G), and cauda (D, H). MYO6 is stained in *red*, F-actin is stained in *green*, and chromatin is stained in *blue*. *Ed*, efferent duct; *Is*, initial segment; *Cap*, caput; *Cor*, corpus; *Cau*, cauda. *Arrows* show the apical region of epithelial cells at the base of microvilli (A–D). Scale bar 50 μm. Higher magnifications of selected image fragments are shown as Zoom images. *Stars* marked epithelial nonciliated cells in *Ed* (A) and epithelial principal cells in different segments of epididymis (B-D); *arrow heads* point to epithelial ciliated cells in *Ed* (A). Scale bar 10 μm. Number of mice analyzed: n = 5 (*Cap*), n = 3 (*Cor* and *Cau*). Graphs (I) represent fluorescence levels in epididymal epithelium of individual segments in *sv*/+ mice, normalized to the *sv*/*sv* mutant background. Error bars represent standard deviation, and statistical significance is indicated as ^*^P ≤ 0.05, ^**^P ≤ 0.01, and ^****^P ≤ 0.0001.

We next evaluated the distributions of MYO6 and F-actin in the following segments of the mouse epididymis: efferent duct-epididymal junction, initial segment, caput, corpus, and cauda (the same zones were studied in all subsequent immunodetection experiments using different antibodies). Using immunocytochemistry, phalloidin staining, and confocal microscopy we detected endogenous MYO6 in all the examined segments in control *sv*/+ mice (Figure 3A–D). This protein was predominantly localized to the apical region of epithelial cells at the base of microvilli (Figure 3A–D, *arrows*), but was also detected scattered throughout their cytoplasm. The strongest MYO6 signal was found in epithelial cells lining the lumen of the common efferent tubule (Figure 3A), particularly in nonciliated cells characterized by an apical brush border of microvilli (Figure 3A, *stars* in Zoom). In contrast, in the apical zone of the efferent tubule epithelial cells, which showed a significantly weaker F-actin staining, a weak fluorescence signal for MYO6 was observed (Figure 3A, *arrow heads* in Zoom). These cells probably correspond to ciliated cells. A strong fluorescence signal of MYO6 was also revealed in the epithelial principal cells of the caput, corpus, and cauda segments of the epididymis (Figure 3B–D, *stars* in Zoom). Finally, no specific MYO6 immunolabeling was detected in the epididymal epithelium of Snell’s waltzer males (Figure 3E–H). These findings were confirmed multiple times in cryo-sections (additional representative immunofluorescence images are shown in Supplementary Figure S2) and by statistical analysis of the fluorescence intensity (Figure 3I and Supplementary Figure S2). We therefore conclude that the microvilli-forming epithelial cells of the epididymis and the common efferent duct express MYO6, with the protein level decreasing in the distal segments of the epididymis.

### Immunodetection of clathrin and the clathrin adaptor protein Dab2 in the epididymal epithelium of control and KO mice

**Figure 4:**
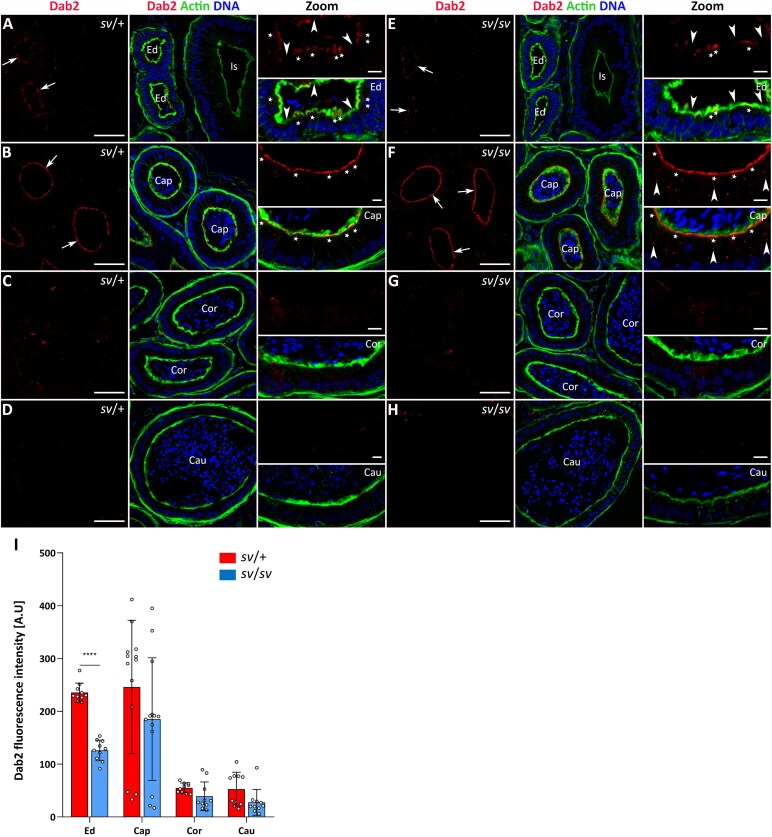
Immunolocalization of Dab2 in the epididymal epithelium of control mice (A-D) and Snell’s waltzer mutants (E–H), and statistical analysis of the fluorescence intensity (I). The following panels show: the common efferent duct and initial segment (A, E), caput (B, F), corpus (C, G), and cauda (D, H). Dab2 is stained in *red*, F-actin is stained in *green*, and chromatin is stained in *blue*. *Ed*, efferent duct; *Is*, initial segment; *Cap*, caput; *Cor*, corpus; *Cau*, cauda. *Arrows* show the apical region of epithelial cells at the base of microvilli (A, B, E, F). Scale bar 50 μm. Higher magnifications of selected image fragments are shown as Zoom images. *Stars* marked epithelial nonciliated cells in *Ed* (A, E) and epithelial principal cells in *Cap* (B, F); *arrow heads* point to epithelial ciliated cells in *Ed* (A, E) and fluorescent spots throughout the cytoplasm of the epithelial principal cells (F). Scale bar 10 μm. Number of mice analyzed: n = 4 (*Cap*), n = 3 (*Cor* and *Cau*). Graphs (I) represent differences in fluorescence levels in individual segments of in control and mutant mice. Error bars represent standard deviation, and statistical significance is indicated as ^****^P ≤ 0.0001.

Since our co-immunoprecipitation experiments confirmed the interaction of MYO6 with the clathrin adaptor Dab2 in mouse epididymis, we further focused on the localization patterns of Dab2 and clathrin in the epididymal epithelium of control and Snell’s waltzer mice. As we expected, Dab2 is present in the epithelium, with the strongest immunofluorescence signal occurring in the apical zone of the epithelial cells in the caput (Figure 4B, F, *arrows*), as well as the common efferent duct (Figure 4A, E, *arrows*). In control males, the Dab2 signal was limited to the apical zone of principal cells (in the caput; Figure 4B, *stars* in Zoom) and nonciliated cells (in the efferent duct; Figure 4A, *stars* in Zoom) with dense microvilli, whereas the ciliated cells in the efferent duct, characterized by weak F-actin fluorescence, showed almost undetectable Dab2 staining (Figure 4A, *arrow heads* in Zoom). Therefore, we conclude that Dab2 colocalizes predominantly with MYO6 in the proximal segment of the mouse epididymis (including the efferent duct) and these both proteins are concentrated in the apical zone of epithelial cells forming a brush border. Next, we decided to determine whether the loss of MYO6 affects distribution of Dab2 in the epithelium of *sv*/*sv* mutant. Indeed, we observed differences in the immunolocalization pattern of this protein in epithelial cells not expressing MYO6. In *sv*/*sv* males, the Dab2 signal was partially lost, especially in the efferent duct (compare Figures 4A and 4E, *stars* in Zoom). In the caput region, in addition to the typical apical localization of Dab2 in the principal epithelial cells (Figure 4F, *stars* in Zoom), numerous fluorescent spots were also observed throughout their cytoplasm (Figure 4F, *arrow heads* in Zoom). We found no significant differences in Dab2 immunodetection in the corpus (Figure 4C, G) and cauda (Figure 4D, H) of the epididymis between control and MYO6 mutant mice. These findings were confirmed multiple times in cryo-sections (additional representative immunofluorescence images are shown in Supplementary Figure S3) and by statistical analysis of the fluorescence intensity (Figure 4I and Supplementary Figure S3).

**Figure 5:**
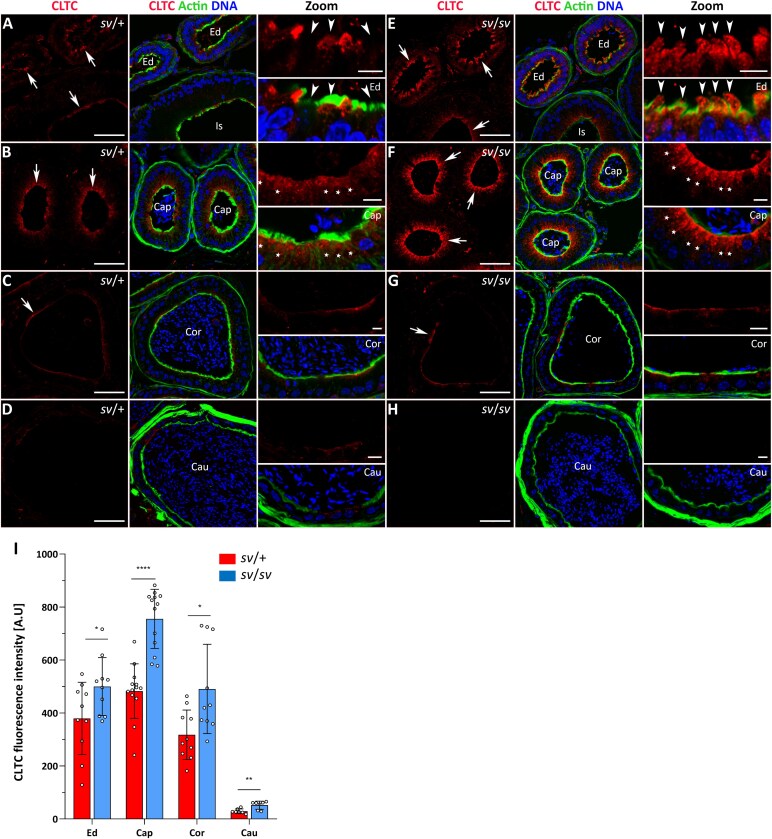
Immunolocalization of clathrin in the epididymal epithelium of control mice (A–D) and Snell’s waltzer mutants (E–H), and statistical analysis of the fluorescence intensity (I). The following panels show: the common efferent duct and initial segment (A, E), caput (B, F) corpus (C, G), and cauda (D, H). Clathrin is stained in *red*, F-actin is stained in *green*, and chromatin is stained in *blue*. *Ed*, efferent duct; *Is*, initial segment; *Cap*, caput; *Cor*, corpus; *Cau*, cauda. *Arrows* show the apical region of epithelial cells at the base of microvilli (A–C, E–G). Scale bar 50 μm. Higher magnifications of selected image fragments are shown as Zoom images. *Stars* marked the principal cells in the caput (B, F), *arrow heads* point to epithelial cells of the efferent duct exhibiting a weak clathrin labeling in *sv*/+ cells (A) and a strong clathrin labeling in *sv*/*sv* cells (E), including membrane protrusions extending into the tubule lumen. Scale bar 10 μm. Number of mice analyzed: n = 5 (*Cap*), n = 3 (*Cor* and *Cau*). Graphs (I) represent differences in fluorescence levels in individual segments of in control and mutant mice. Error bars represent standard deviation, and statistical significance is indicated as ^*^P ≤ 0.05, ^**^P ≤ 0.01, and ^****^P ≤ 0.0001.

During clathrin-mediated endocytosis, MYO6 plays a crucial role in stabilizing the endocytic machinery, supporting the formation of clathrin-coated pits and facilitating the intracellular distribution of newly formed vesicles [2]. To verify the potential role of MYO6 in this process in the mouse epididymis, we next addressed the localization of clathrin in the epididymal epithelium of control *sv*/+ mice and *sv*/*sv* mutants. Our data showed that clathrin is abundant in the proximal segments of the mouse epididymis, primarily in the caput (Figure 5B, F  *arrows*) and the common efferent duct (Figure 5A, E  *arrows*), where both MYO6 and Dab2 were mainly localized. We also observed fluorescence signal for clathrin in the epididymal epithelium of the corpus (Figure 5C, G  *arrows*), whereas a substantially lower signal was detected in the cauda (Figure 5D, H). In both control and MYO6 mutant mice, clathrin was strongly concentrated in the apical region of the principal cells of the caput, diffusing toward the cell interior (Figure 5B, F  *stars* in Zoom). In contrast, the pattern of clathrin localization in efferent duct epithelial cells of *sv*/+ mice was different compared to *sv*/*sv* mice. While in the control group not all epithelial cells showed strong clathrin staining (Figure 5A, *arrow heads* in Zoom), the MYO6 mutant displayed clathrin accumulation throughout the efferent duct epithelium, with an intense fluorescence signal present in membrane protrusions extending into the tubule lumen (Figure 5E, *arrow heads* in Zoom). These findings were confirmed multiple times in cryo-sections (additional representative immunofluorescence images are shown in Supplementary Figure S4) and by statistical analysis of the fluorescence intensity (Figure 5I and Supplementary Figure S4). We therefore conclude that MYO6 depletion leads to impaired distribution of clathrin and the clathrin adaptor protein Dab2 in epithelial cells lining the tubules of the proximal epididymal segments and the common efferent duct.

### Immunodetection of the adaptor proteins GIPC1 and APPL1 in the epididymal epithelium of control and KO mice

**Figure 6:**
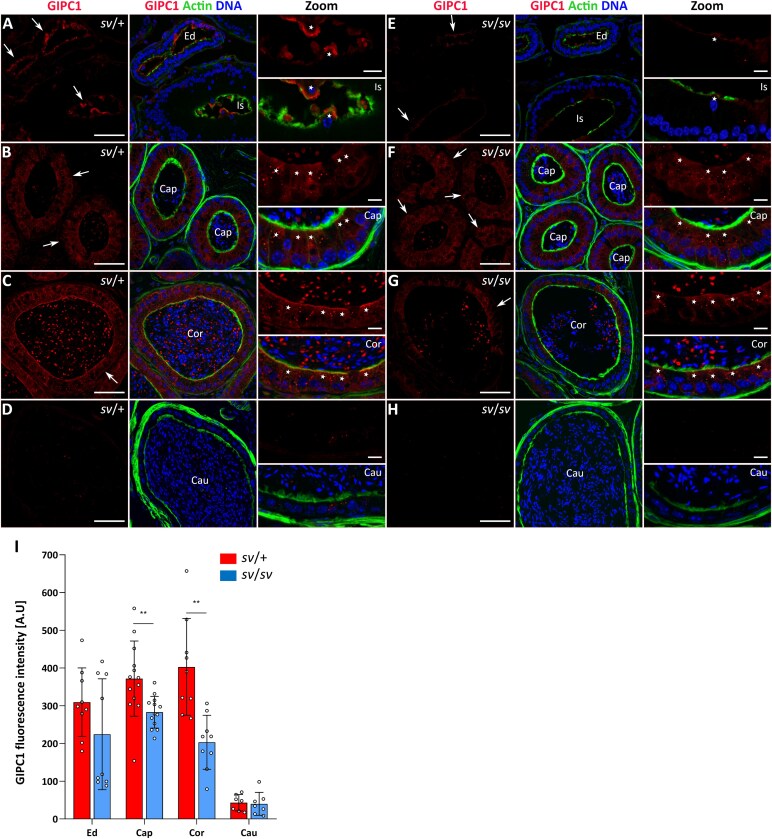
Immunolocalization of GIPC1 in the epididymal epithelium of control mice (A–D) and Snell’s waltzer mutants (E-H), and statistical analysis of the fluorescence intensity (I). The following panels show: the common efferent duct and initial segment (A, E), caput (B, F) corpus (C, G), and cauda (D, H). GIPC1 is stained in *red*, F-actin is stained in *green*, and chromatin is stained in *blue*. *Ed*, efferent duct; *Is*, initial segment; *Cap*, caput; *Cor*, corpus; *Cau*, cauda. *Arrows* show the tubules of *Is*, *Cap*, and *Cor* (A–C, E–G). Scale bar 50 μm. Higher magnifications of selected image fragments are shown as Zoom images. *Stars* marked epithelial cells/protrusions in *Is*, *Cap*, and *Cor* (A–C, E–G). Scale bar 10 μm. Number of mice analyzed: n = 5 (*Cap*), n = 3 (*Cor* and *Cau*). Graphs (I) represent differences in fluorescence levels in individual segments of in control and mutant mice. Error bars represent standard deviation, and statistical significance is indicated as ^**^P ≤ 0.01.

Given that MYO6 is recruited to APPL1-positive early endosomes through interaction with GIPC1 [15, 16], and that we confirmed the presence of MYO6-GIPC1 complexes in the mouse epididymis, we next labeled cryo-sections of mouse epididymal tubules with antibodies to GIPC1 and APPL1. Immunofluorescent analysis indicated the presence of GIPC1 in almost all epididymal segments, with the strong signal coming from the common efferent duct to the corpus in control mice (Figure 6A–C, *arrows*). In the initial segment, a prominent accumulation of GIPC1 was detected in the epithelial cell protrusions extending into the lumen (Figure 6A, *stars* in Zoom), whereas a more homogeneous GIPC1 labeling, from the apical zone throughout the cytoplasm, was present in epithelial cells of the caput (Figure 6B, *stars* in Zoom) and corpus (Figure 6C, *stars* in Zoom). Furthermore, characteristic labeling was found in the lumen of the tubules (especially in the corpus segment), with a punctate accumulation of GIPC1 between maturing sperm (Figure 6B, C). We next explored whether the absence of MYO6 has an impact on the localization pattern of this adaptor protein. Our results indicate a partial loss of GIPC1 labeling in the epithelium of the efferent duct (Figure 6E, *arrows*) as well as in the caput (Figure 6F, *arrows*) and the corpus (Figure 6G, *arrows*) segments in Snell’s waltzer mutants; however, the overall localization pattern of this protein remained comparable to that of control mice across all examined regions. These findings were confirmed multiple times in cryo-sections (additional representative immunofluorescence images are shown in Supplementary Figure S5) and by statistical analysis of the fluorescence intensity (Figure 6I and Supplementary Figure S5).

**Figure 7:**
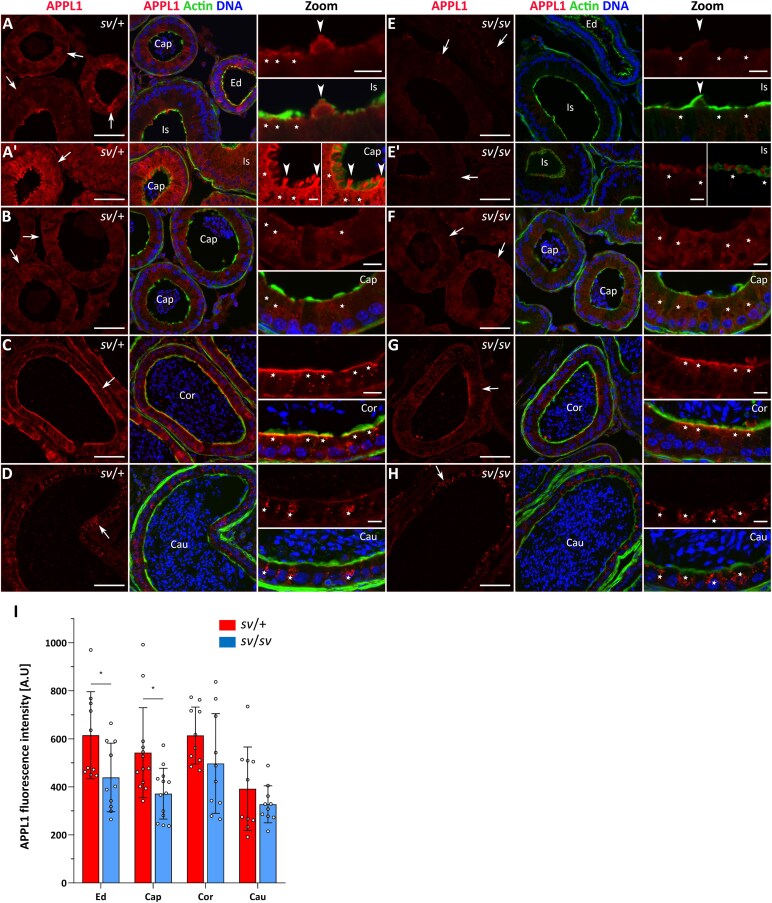
Immunolocalization of APPL1 in the epididymal epithelium of control mice (A–D) and Snell’s waltzer mutants (E–H), and statistical analysis of the fluorescence intensity (I). The following panels show: the common efferent duct and initial segment (A, A’, E, E’), caput (B, F) corpus (C, G), and cauda (D, H). APPL1 is stained in *red*, F-actin is stained in *green*, and chromatin is stained in *blue*. *Ed*, efferent duct; *Is*, initial segment; *Cap*, caput; *Cor*, corpus; *Cau*, cauda. *Arrows* show the tubules in *Ed*, *Is*, *Cap*, *Cor*, and *Cau* (A–D, E–H). Scale bar 50 μm. Higher magnifications of selected image fragments are shown as Zoom images. *Stars* marked principal cells in *Is*, *Cap*, *Cor*, and *Cau* (A–D, E–H), and *arrow heads* point to epithelial cells/protrusions (Figure 7A, A’, E). Scale bar 10 μm. Number of mice analyzed: n = 4. Graphs (I) represent differences in fluorescence levels in individual segments of in control and mutant mice. Error bars represent standard deviation, and statistical significance is indicated as ^*^P ≤ 0.05.

Since the MYO6-GIPC1 complex associates with the APPL1-positive endosomes [15, 16], we next performed immunolocalization of this endosome marker in the epididymal epithelium of control and Snell’s waltzer mice. As shown in Figure 7, APPL1 was detected throughout the entire epididymal epithelium of *sv*/+ males, including the common efferent duct (Figure 7A–D, *arrows*). In general, similar to GIPC1, APPL1 distribution included the cytoplasm of epithelial cells from the apical membrane forming microvilli to their basal zone. However, the APPL1 localization pattern was particularly dynamic in the proximal segments of the epididymis – the initial segment and caput – where tubules with lower or higher levels of APPL1 fluorescence were observed (compare Figure 7A and A’). In the initial segment, a dominant apical enrichment of APPL1 in the principal cells was found at the base of microvilli (Figure 7A, *stars* in Zoom), whereas in epithelial cells showing weak F-actin fluorescence (probably clear cells), this protein enriched the apical zone of their cytoplasm that forms a bulge towards the tubule lumen (Figure 7A, *arrow head* in Zoom). However, in selected tubules corresponding to the caput, APPL1 accumulated through the cytoplasm of epithelial cells (Figure 7A’), as well as in numerous protrusions extending into the lumen (Figure 7A’, *arrow heads* in Zoom). In the other epididymal segments of control mice, the pattern of APPL1 localization was diverse. The protein was uniformly distributed in epithelial cells of the caput (Figure 7B, *stars* in Zoom) and cauda (Figure 7D, *stars* in Zoom) zones, but was more concentrated at the base of the microvilli in the corpus zone (Figure 7C, *stars* in Zoom). Interestingly, numerous fluorescent spots corresponding to APPL1 aggregates, which may represent endosomes, were present in the cytoplasm of epithelial cells in the caput, corpus, and cauda (Figure 7B–D, Zoom). Finally, reduced APPL1 labeling was observed in the epithelial cells of the efferent duct and the proximal regions of the epididymis in MYO6-deficient mice (compare Figures 7E, E’, F and 7A, A’, B, *arrows*), whereas the APPL1 localization pattern in *sv*/*sv* mutant was comparable across all examined regions to that of control mice (compare Figures 7E-H and 7A-D, *arrows).* These findings were confirmed multiple times in cryo-sections (additional representative immunofluorescence images are shown in Supplementary Figure S6) and by statistical analysis of the fluorescence intensity (Figure 7I and Supplementary Figure S6). We therefore conclude that MYO6 depletion impaired the distribution of the adaptor proteins GIPC1 and APPL1 in epithelial cells lining the tubules of the proximal epididymis in mouse.

It should be emphasized that the immunoblotting verified the specificity of all the commercial primary antibodies used during the studies, and the negative control without the primary antibodies confirmed the specificity of the fluorochrome-labeled secondary antibody (Supplementary Figure S7).

### Ultrastructural examination of the apical domain of epididymal epithelial cells in control and Snell’s waltzer mice

**Figure 8:**
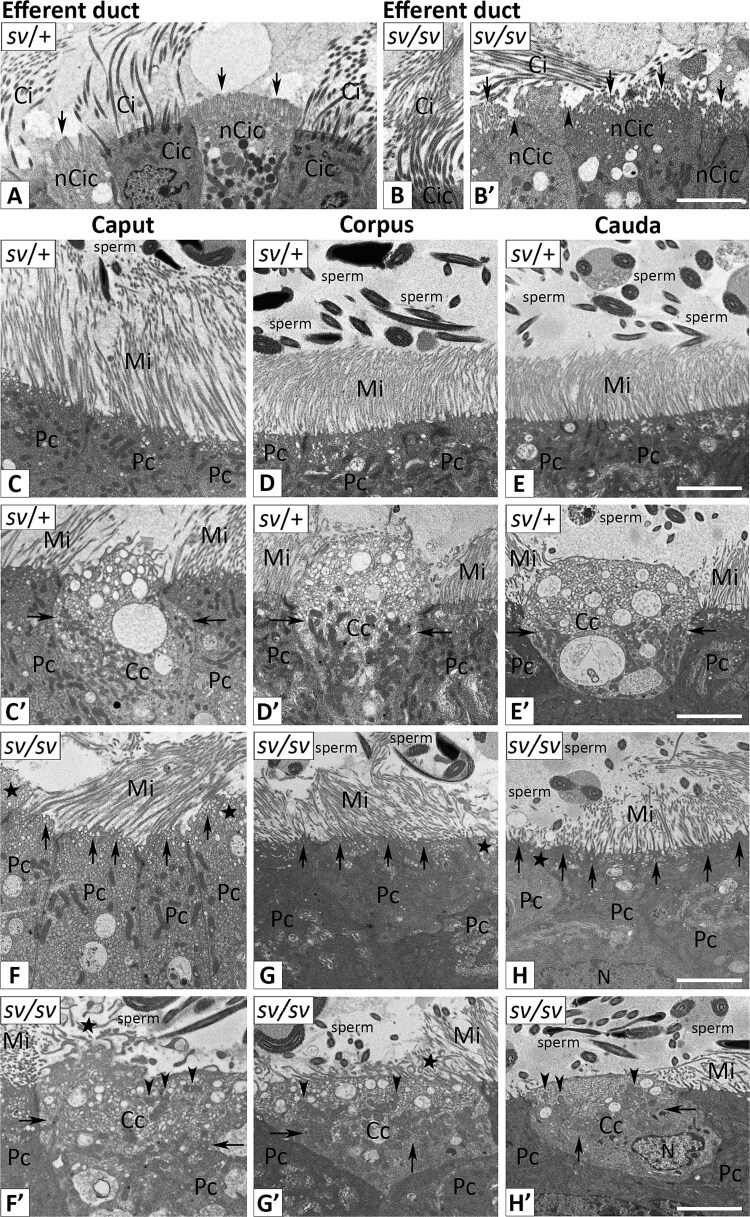
Transmission electron microscopy of the apical epithelial zone of the common efferent duct and the epididymis in control (*sv*/+) and MYO6 mutant (*sv*/*sv*) male mice. Panel A (control) and B-B′ (MYO6 mutant) show the ultrastructure of the apical epithelial zone of the efferent duct. *Arrows* and *arrow heads* point to brush border structure in the nonciliated cells. Panels C–E (control) and F–H (MYO6 mutant) show the ultrastructure of the apical zone of principal cells containing microvilli, whereas panels C′–E’ (control) and F′–H′ (MYO6 mutant) show the ultrastructure of clear cells in the following epididymal segments: caput, corpus, and cauda. *Arrows* indicate: the basal zone of clear cells rich in mitochondria in control mice (C′–E’), defects in the apical membrane of principal cells in MYO6 mutant (F–H), and mitochondria in the basal zone of clear cells in MYO6 mutant (F′–H′). *Arrow heads* indicate mitochondria in the apical zone of clear cells in MYO6 mutant (F′–H′). *Stars* indicate: disappearance of microvilli in the apical zone of principal cells (F–H) and abnormal microvilli formed in the apical zone of clear cells in MYO6 mutant (F′–G’). *Cc*, clear cells; *CiC*, ciliated cells; Mi, microvilli; *nCiC*, nonciliated cells; *Pc*, principal cells. Scale bar 5 μm.

MYO6 is essential for the proper formation and functioning of brush border structures in highly specialized epithelia, as its deficiency in MYO6 mutant mice causes various ultrastructural defects of microvilli and stereocilia [8, 21–26, 29, 31]. To determine whether the absence of MYO6 in the mouse epididymis is associated with structural alterations in polarized epithelial cells, we performed comparative ultrastructural analysis using ultrathin cross-sections of *sv*/+ and *sv*/*sv* epididymal tubules and transmission electron microscopy. In our studies, we paid particular attention to two types of epididymal epithelial cells, principal and clear cells, which represent the dominant cell types throughout the length of the epididymis. However, we first examined the ultrastructure of the epithelial cells lining the common efferent duct, as this region displayed the highest MYO6 expression. The efferent duct tubules are lined by an epithelium composed of two principal cell types: ciliated and nonciliated cells [43, 44]. Ciliated cells possess motile cilia that project into the lumen between microvilli and facilitate sperm transport through the duct, whereas nonciliated cells display an apical brush border of microvilli that mediates absorptive functions. Indeed, in the efferent duct epithelium of control mice (*sv*/+), we confirmed the presence of both cell types bearing properly developed cilia (Figure 8A and Supplementary Figure S8A’) or microvilli (Figure 8A, *arrows*, and Supplementary Figure S8A, A’, *arrows*). In MYO6 mutant, we observed pronounced defects in brush border formation in nonciliated cells (Figure 8B’, *arrows*/*arrow heads*). However, it should be emphasized that these defects were not always so obvious, because in *sv*/*sv* males we also observed properly formed microvilli in nonciliated cells (Supplementary Figure S8B’, *arrow*). We then analyzed the ultrastructure of the apical epithelial domain of the mouse epididymis. As shown in Figure 8C–E, in control males the apical surface of the principal cells is covered with tightly packed microvilli that form a brush border and are long in the caput, then become progressively shorter towards the cauda. In the apical cytoplasm of these cells, numerous small vesicles were observed, along with densely packed mitochondria. In contrast, *sv*/*sv* principal cells have an irregular brush border where the apical membrane is no longer pulled down to the very base of the microvilli (Figure 8F–H, *arrows*). Such membrane tethering defect was observed in all epididymal segments examined. Furthermore, microvilli varied in length within the same epididymal segment and were often bent compared with the control group. Interestingly, areas devoid of microvilli were observed in multiple locations on the apical membrane of principal cells obtained from MYO6 mutant mice (Figure 8F–H, *stars*). Clear cells, which lacked microvilli or had only a few, displayed a highly organized intracellular architecture in *sv*/+ males (Figure 8C’–E’). Their cytoplasm was divided into two distinct regions: an apical zone containing an accumulation of smaller vesicles, and a mitochondria-enriched basal region with much larger vesicles. Vesicles of various sizes also accumulated in *sv*/*sv* clear cells (Figure 8F’–H’). However, the two-zone spatial organization of the cytoplasm of these cells was disrupted in MYO6 mutant, as vesicles were uniformly distributed throughout the cytoplasm, as were mitochondria, whose presence was observed in both the basal (Figure 8F’–H′, *arrows*) and apical (Figure 8F’–H′, *arrow heads*) zones of clear cells. Thus, we observed loss of the apical “vesicular zone” in *sv*/*sv* clear cells, but also abnormal cell membrane protrusions on the surface of these cells (Figure 8F’, G’, *stars*). These findings were confirmed multiple times in the ultrathin sections (Supplementary Figure S8). Taken together, we conclude that MYO6 deficiency in the mouse epididymis results in disturbed ultrastructure of epididymal epithelial cells, which may have a negative impact on their function in sperm maturation.

## Discussion

In this work, we demonstrate that both shorter and longer splice variants of MYO6 are expressed in mouse epididymis and confirm MYO6 interactions with the adaptor proteins Dab2 and GIPC1. Our results indicate that MYO6 participates in clathrin-mediated endocytosis in epididymal epithelial cells and plays a role in the structural organization of microvilli. Several defects revealed in MYO6-deficient mice support this conclusion. To the best of our knowledge, this study provides the first evidence for a physiological role of myosin MYO6 in the polarized epithelial cells of the mouse epididymis.

### Diversity of MYO6 splice variants in the mouse epididymis

Our findings demonstrate a segment-specific expression of MYO6 splice variants in the mouse epididymis, with both short and long isoforms expressed in the caput and corpus, and the MYO6-NI variant predominating in the cauda. This pattern correlates with the cellular diversity of the epididymal epithelium, where polarized, microvilli-rich cells coexist with non-polarized cells that differ in endocytic activity. In contrast to the seminiferous tubules of the mouse testis, where only short MYO6 isoforms have been detected [34], our present data show that isoforms containing the LI insert are also expressed in the epididymis. These splice variants are typically associated with polarized cells and clathrin-coated structures at the apical surface, whereas NI and SI variants function predominantly in cells lacking microvilli, supporting their role in cargo sorting and early endocytic trafficking cortex [51]. This suggests that alternative splicing contributes to the recruitment of MYO6 to distinct vesicle populations depending on cell type and functional diversity. Previous studies have reported divergent results regarding MYO6 splice variant expression and function across cell types. The LI isoform in polarized, microvilli-rich cells (such as NRK fibroblasts and Caco2) associates with clathrin-coated structures [51], whereas in nonpolarized ARPE-19 cells, MYO6-LI does not colocalize with clathrin or AP-2 and instead facilitates translocation of uncoated endocytic vesicles via GIPC1 [52, 53]. The NI isoform shows reduced targeting to clathrin-coated structures in nonpolarized cells [51, 52], yet both LI and NI splice variants can bind the endocytic protein Dab2 [13]. Loss of MYO6-NI in Snell’s waltzer fibroblasts impairs clathrin-mediated endocytosis [54]; this work demonstrated that short MYO6 isoforms can be expressed in nonpolarized cells, where they regulate early apical endocytosis. However, Dance et al. [53] showed that MYO6, regardless of splice form, associates with uncoated endocytic vesicles in all examined cell types, indicating that the LI insert is not strictly required for targeting to clathrin-coated pits. Nonpolarized MDCK cells express MYO6-NI isoform, while polarized cells express NI, LI, and L + S splice variants, with MYO6-LI localized predominantly to the apical domain and colocalizing with actin [55]. These findings suggest that while alternative splicing can influence MYO6 localization and function, it is not universally determinant; adaptor interactions may play a more critical role in regulating MYO6 activity. In the mouse epididymis (including the efferent duct), MYO6 isoforms are differentially expressed along the tubule and preferentially bind different adaptor proteins in defined regions, likely supporting segment-specific epididymal functions. However, further structural, functional, and single-molecule studies are needed to clarify the mechanisms and biological roles of individual MYO6 splice variants in the epididymis.

### MYO6 is involved in clathrin-mediated endocytosis in mouse epididymal epithelium

Using immunocytochemistry and confocal microscopy, endogenous MYO6 was detected throughout the epididymis of control *sv*/+ mice, with highest accumulation in epithelial cells of the common efferent duct and caput, which possess an apical brush border. In these cells, MYO6 accumulated in the apical region at the base of microvilli, a pattern similar to other mammalian epithelia with well-developed microvilli, including the intestinal epithelium [21, 22, 24–26], renal proximal tubule [8, 20], and cochlear hair cells [6, 27–29]. In cochlear hair cells, MYO6 concentrates in the actin-rich cuticular plate at the base of stereocilia, and its loss leads to stereocilia disorganization and hair cell degeneration, suggesting a role in anchoring the apical membrane and maintaining endocytic compartment integrity [6, 27, 29–32]. In the intestinal and renal proximal tubule epithelia, MYO6 localizes to the inter-microvillar and subapical/terminal web regions, with lower levels at basolateral membranes [8, 20–22, 24–26]. Here, MYO6 regulates clathrin-mediated endocytosis, facilitating protein uptake in enterocytes, megalin receptor internalization in the kidney, and the trafficking of CFTR, NHE3, and sodium transporters into clathrin-coated pits [8, 20–22, 24–26, 56]. Loss of MYO6 function in Snell’s waltzer mice leads to defective apical endocytosis and delayed uptake of markers such as horseradish peroxidase, highlighting its essential role in vesicle trafficking in polarized epithelial cells [8, 21–26]. We show that MYO6, together with Dab2 and clathrin, is predominantly localized in the common efferent duct and proximal epididymal segments, concentrated in the apical region of epithelial cells forming a brush border. Moreover, we confirm that the longer MYO6 isoforms (containing LI) are preferentially expressed in the caput and corpus, with LI facilitating recruitment to clathrin-coated pits via Dab2 and the clathrin-binding domain [13, 14]. Nonciliated epithelial cells of the efferent duct absorb most luminal fluid [43, 44], while principal cells, primarily secretory, also participate in receptor-mediated endocytosis [39]. In MYO6-deficient mice, Dab2 and clathrin distribution is disrupted in these epithelial cells, with clathrin abnormally accumulated in the cytoplasm and membrane protrusions, indicating endocytic dysfunction. Thus, we propose that MYO6 isoforms containing the LI splice insert participate in the early stages of endocytosis within the apical region of epididymal epithelial cells in the proximal segments and are recruited to clathrin-coated pits and vesicles through interactions with Dab2 and the clathrin-binding domain located in the distal tail of MYO6.

Several data have shown that MYO6 isoforms lacking LI preferentially localize to uncoated early endosomes via interaction with GIPC1 through the RRL motif [15, 16]. We observed MYO6-NI splice variant expression throughout the epididymis, predominating in distal segments, with GIPC1 localized primarily to the epithelium of the caput and corpus, and APPL1-positive early endosomes detected in all segments. This suggests that short MYO6 splice variants may mediate early endosome trafficking via GIPC1 in proximal segments, whereas in the distal segment, other interaction partners are likely involved. Supporting this, in the testis, MYO6-SI and GIPC1 localize to actin-rich tubulobulbar complexes involved in endocytosis of intercellular junctions, and MYO6 loss disrupts actin organization and early endocytic compartments [36]. Overall, our data indicate that MYO6 is an important component of endocytic protein complexes in mouse epididymal epithelium, participating at multiple stages of endocytosis. Its functions include recruitment to clathrin-coated pits, regulation of early endosome trafficking, and structural roles in maintaining actin organization and tethering of membrane compartments to the cytoskeleton.

Principal cells are the predominant cell type throughout the epididymis, particularly active in protein biosynthesis and secretion in proximal segments [39]. In the caput, we confirmed the expression of short MYO6 isoforms, which may function in the secretory pathway, unlike LI-containing splice variants of MYO6. Several studies support a role for MYO6 in exocytosis. MYO6 localizes to the Golgi apparatus, the ruffling leading edge, and a cytosolic pool in fibroblasts [17], and Snell’s waltzer fibroblasts show reduced Golgi size and exocytic defects, indicating its involvement in Golgi organization and function [17, 18]. MYO6 also binds otoferlin in sensory hair cells, suggesting a role in synaptic exocytosis [57, 58], and the MYO6-SI isoform tethers secretory granules to the actin cortex in neurosecretory cells [19]. Consistently, we recently found that MYO6 splice variants lacking LI are expressed in mouse testes and localize to the spermatid Golgi complexes and nascent acrosome, and MYO6 deficiency disrupts Golgi structure and secretory granules in Snell’s waltzer testes [35]. Further studies using secretory markers as well as detailed ultrastructural analyses are needed to verify the potential role of MYO6 in the secretory activity of epididymal epithelial cells.

### MYO6 is crucial in the structural organization of polarized mouse epididymal epithelium

MYO6 plays a key role in the formation and maintenance of brush border structures in polarized epithelia, as its deficiency causes ultrastructural defects in microvilli and stereocilia [8, 21–26, 29, 31]. The most severe phenotypes occur in cochlear hair cells, where stereocilia degeneration leads to deafness [29, 31]. Similar defects are observed in intestinal and renal epithelia, including shortened, fused, or irregular microvilli and disorganized terminal webs [8, 21–24]. Our current results are consistent with these previous reports. In the epididymal epithelium of *sv*/*sv* mice, impaired attachment of microvilli to the apical membrane of principal cells was observed across all segments, various microvilli defects, and in some cases their partially loss. Similar ultrastructural anomalies were observed in nonciliated cells of the common efferent duct in MYO6-deficient males. Moreover, light vesicles were accumulated in the apical region of the principal cells, particularly in the caput, while clear cells showed loss of the apical vesicular zone and abnormal membrane protrusions. It should be noted that clear cells, enriched in the cauda, possess extensive endocytic machinery [39]. Therefore, the defects observed in the ultrastructure of these cells in *sv*/*sv* males may indicate an impairment of the endocytic process. In other mammalian polarized epithelia, MYO6 localizes to the base of microvilli/stereocilia and between the actin core and lateral membrane [8, 20–22, 24, 27, 30, 59, 60], suggesting it anchors the apical membrane to the actin-rich zone. Its absence may allow the apical membrane to pull up between microvilli, causing brush border defects. This anchoring role complements MYO6 function in membrane trafficking during endocytosis at the base of microvilli. Overall, our data provide the first evidence that MYO6 deficiency in the mouse epididymis as well as in the common efferent duct leads to severe ultrastructural defects, likely impairing epithelial function.

## Conclusions

Our results demonstrate that distinct MYO6 splice variants are expressed in the mouse epididymis, interacting with adaptor proteins Dab2 and GIPC1. Their distribution, together with clathrin and the early endosomal marker APPL1, indicates that MYO6 participates in multiple stages of clathrin-mediated endocytosis in epididymal epithelial cells. MYO6 deficiency in Snell’s waltzer mice disrupts the localization of endocytic markers and causes ultrastructural defects in apical microvilli and cytoplasmic vesicles, highlighting its role in maintaining epithelial polarity and intracellular organization. This study provides the first evidence of MYO6 expression in polarized epithelial cells of the mammalian epididymis. Further functional work is needed to elucidate the molecular mechanisms underlying its role in endocytic trafficking and epithelial architecture in the mouse epididymis.

## Supplementary Material

Supplementary_Figures_S1-S8_ioag031

Supplementary_Tables_S1-S4_ioag031

## Data Availability

The data underlying this article will be shared on reasonable request to the corresponding author.
