# Supplementary material for: Myosin VI is involved in the structural organization and molecular composition of epididymal epithelial cells in mouse
Source: Biol Reprod. 2026 Feb 2;114(5):1747–64. doi: 10.1093/biolre/ioag031 (PMC13175989; doi:10.1093/biolre/ioag031)
Supplement: Supplementary_Figures_S1-S8_ioag031 [file supplementary_figures_s1-s8_ioag031.pdf]

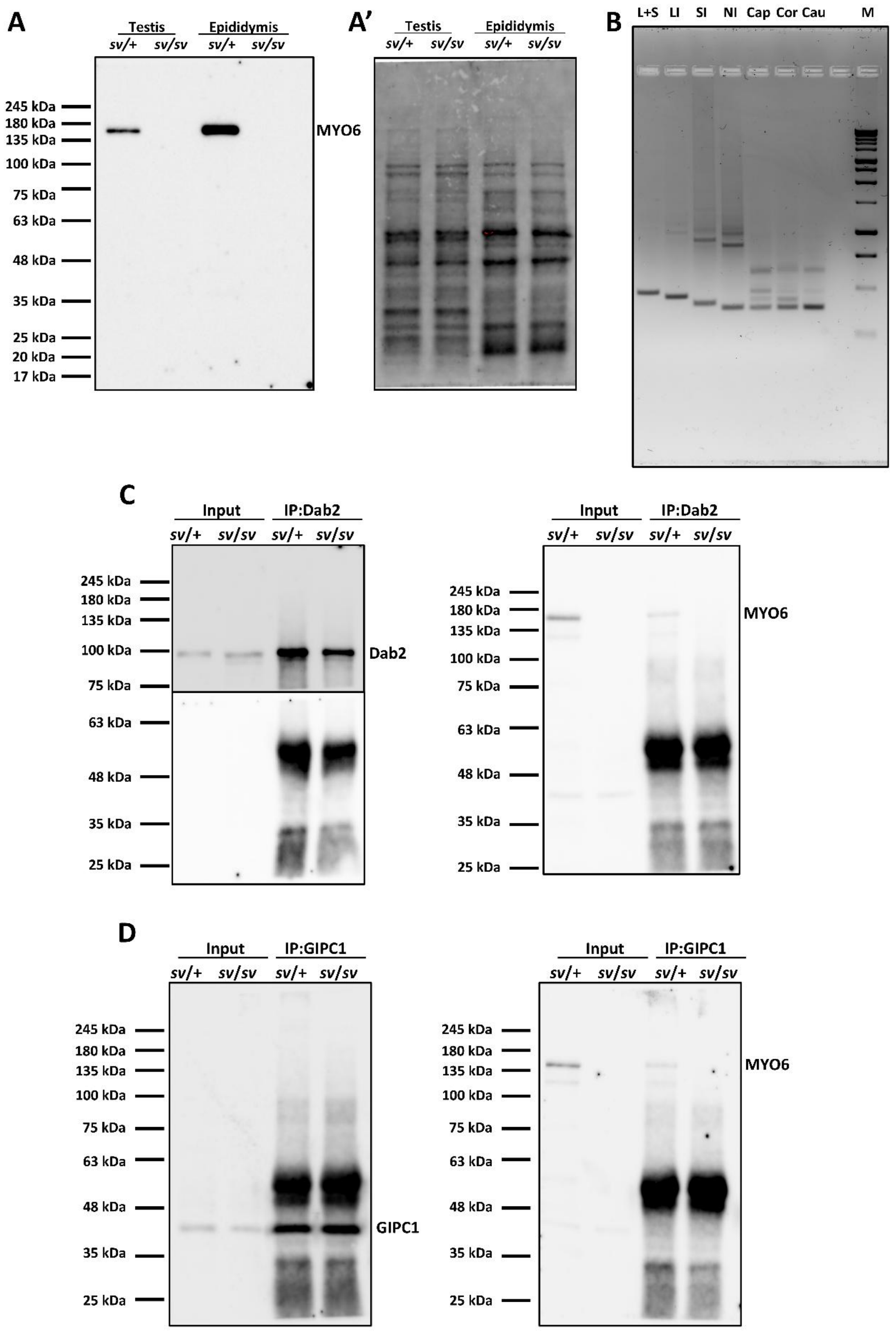

Supplementary Figure S1. Specificity of the anti-MYO6 antibody verified by immunoblotting (**A**); stain-free blot with total protein post-transfer (**A'**). Detection of various MYO6 splice variants in different regions of the epididymis: caput (*Cap*), corpus (*Cor*), and cauda (*Cau*) by RT-PCR (**B**); DNA marker (M) Perfect Plus 1 kb DNA Ladder (EURx). Immunoblotting of inputs and co-immunoprecipitated proteins: MYO6 and Dab2 (**C**), MYO6 and GIPC1 (**D**); the protein marker (Protein Marker VI, 10 – 245 kDa, pre-stained, AppliChem) is indicated on the left of each blot.

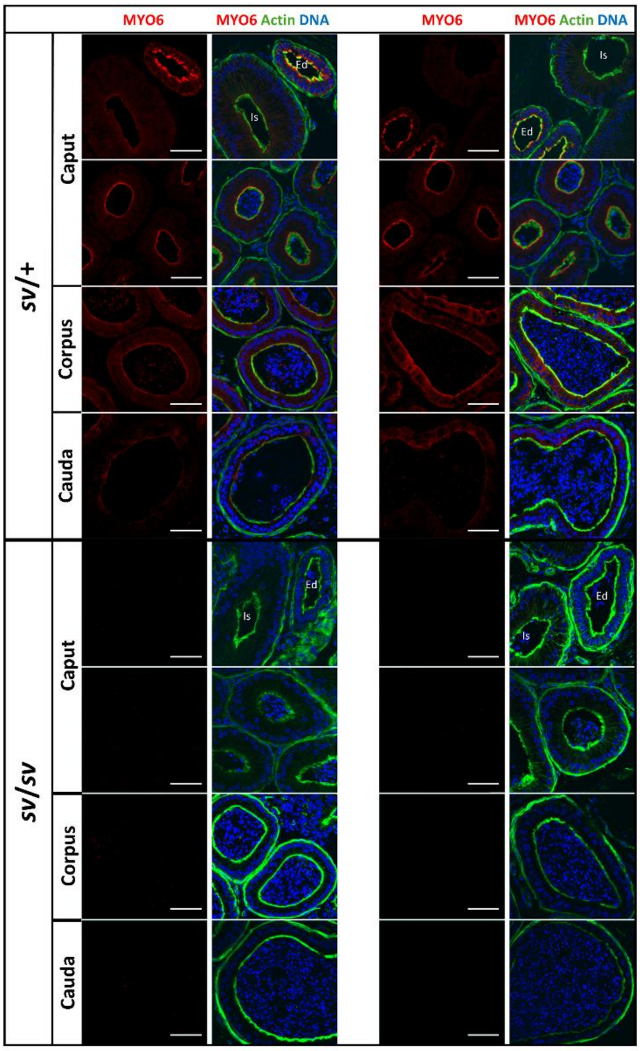

Group		p-values
		MYO6
sv/+	Ed *vs* Cap	0,0001
	Ed *vs* Cor	0,0001
	Ed *vs* Cau	0,0001
	Cap *vs* Cor	0,0045
	Cap *vs* Cau	0,0005
	Cor *vs* Cau	0,0175

Supplementary Figure S2. Immunolocalization of MYO6 in the epididymal epithelium of control mice (*sv*/+) and Snell's waltzer mutants (*sv*/*sv*). The following panels show: caput (including the common efferent duct and initial segment), corpus, and cauda. MYO6 is stained in *red*, F-actin is stained in *green*, and chromatin is stained in *blue*. Scale bar 50 μm. Exact p-values summarized in the table represent differences in fluorescence levels in individual segments (*Ed*, efferent duct; *Is*, initial segment; *Cap*, caput; *Cor*, corpus; *Cau*, cauda) in control and mutant mice.

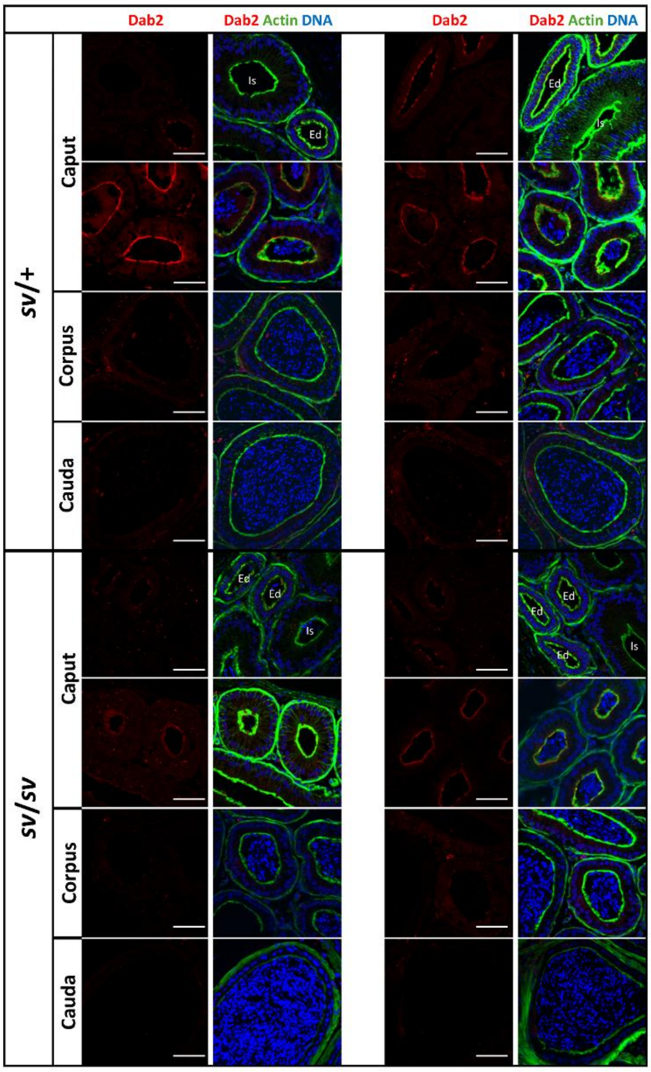

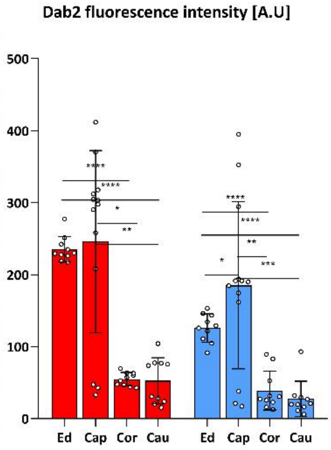

Group		p-values
		Dab2
sv/+	Ed *vs* Cap	0,1475
	Ed *vs* Cor	0,0001
	Ed *vs* Cau	0,0001
	Cap *vs* Cor	0,0178
	Cap *vs* Cau	0,0011
	Cor *vs* Cau	0,844
sv/*sv*	Ed *vs* Cap	0,0303
	Ed *vs* Cor	0,0001
	Ed *vs* Cau	0,0001
	Cap *vs* Cor	0,0032
	Cap *vs* Cau	0,0005
	Cor *vs* Cau	0,2475

Supplementary Figure S3. Immunolocalization of Dab2 in the epididymal epithelium of control mice (*sv*/+) and Snell's waltzer mutants (*sv*/*sv*). The following panels show: caput (including the common efferent duct and initial segment), corpus, and cauda. Dab2 is stained in *red*, F-actin is stained in *green*, and chromatin is stained in *blue*. Scale bar 50 µm. Graphs represent differences in fluorescence levels in individual segments (*Ed*, efferent duct; *Is*, initial segment; *Cap*, caput; *Cor*, corpus; *Cau*, cauda) in control and mutant mice. Error bars represent standard deviation, and statistical significance is indicated as $*p \leq 0.05$, $**p \leq 0.01$, $***p \leq 0.001$ and $****p \leq 0.0001$. Exact p-values are summarized in the table.

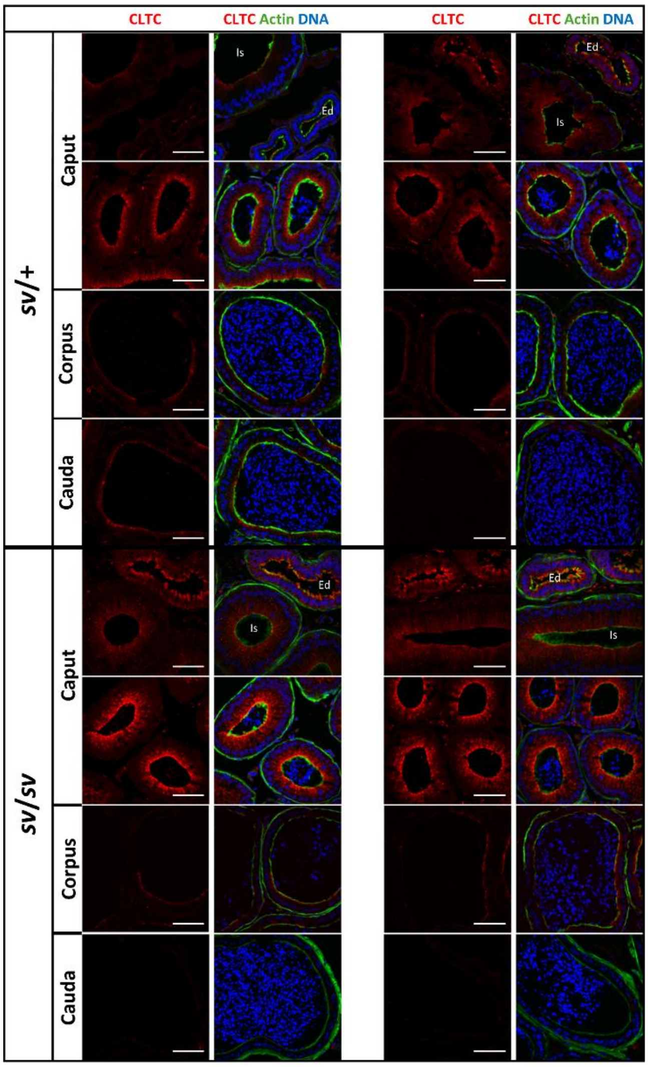

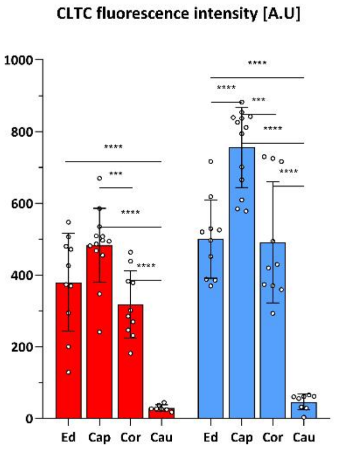

Group		p-values
		CLTC
sv/+	Ed *vs* Cap	0,0575
	Ed *vs* Cor	0,2552
	Ed *vs* Cau	0,0001
	Cap *vs* Cor	0,0006
	Cap *vs* Cau	0,0001
	Cor *vs* Cau	0,0001
sv/*sv*	Ed *vs* Cap	0,0001
	Ed *vs* Cor	0,8886
	Ed *vs* Cau	0,0001
	Cap *vs* Cor	0,0008
	Cap *vs* Cau	0,0001
	Cor *vs* Cau	0,0001

Supplementary Figure S4. Immunolocalization of CLTC in the epididymal epithelium of control mice (*sv*/+) and Snell's waltzer mutants (*sv*/*sv*). The following panels show: caput (including the common efferent duct and initial segment), corpus, and cauda. CLTC is stained in *red*, F-actin is stained in *green*, and chromatin is stained in *blue*. Scale bar 50 µm. Graphs represent differences in fluorescence levels in individual segments (*Ed*, efferent duct; *Is*, initial segment; *Cap*, caput; *Cor*, corpus; *Cau*, cauda) in control and mutant mice. Error bars represent standard deviation, and statistical significance is indicated as ∗∗∗$p \leq 0.001$, ∗∗∗∗$p \leq 0.0001$. Exact p-values are summarized in the table.

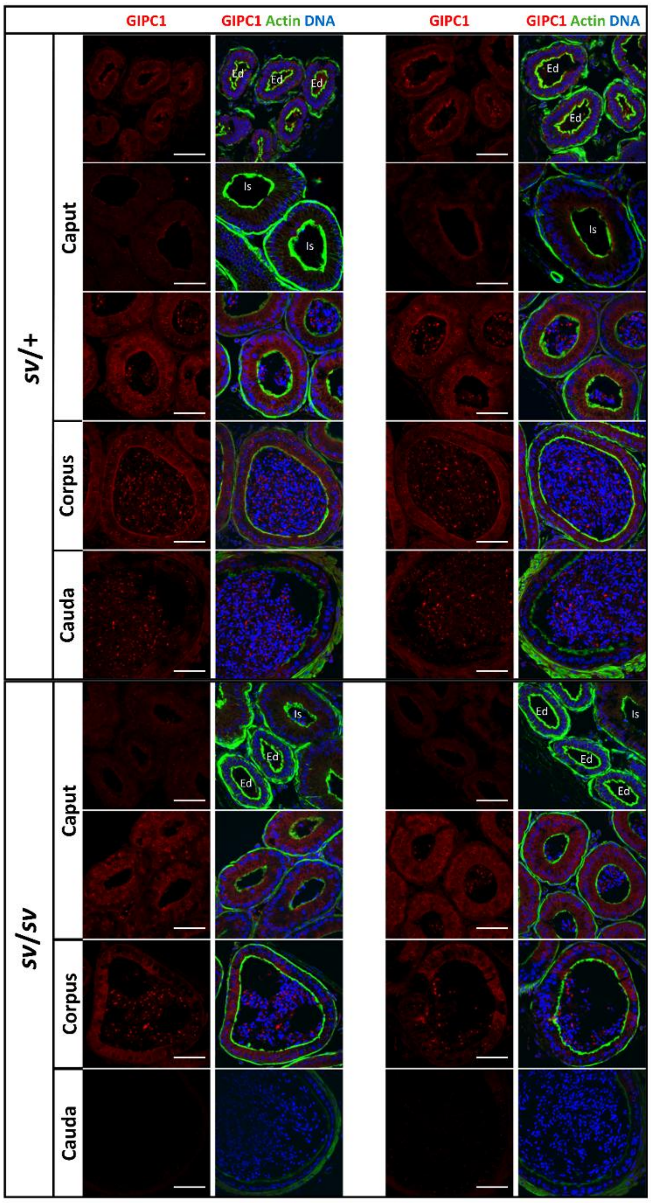

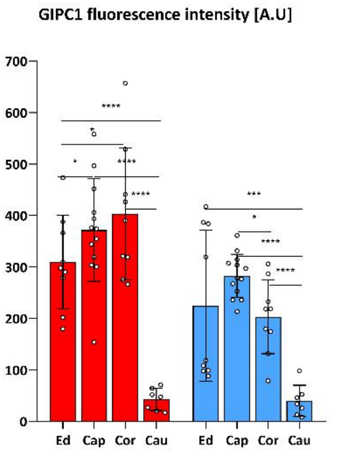

Group		p-values
		GIPC1
sv/+	Ed *vs* Cap	0,0406
	Ed *vs* Cor	0,0407
	Ed *vs* Cau	0,0001
	Cap *vs* Cor	0,5506
	Cap *vs* Cau	0,0001
	Cor *vs* Cau	0,0001
sv/sv	Ed *vs* Cap	0,6005
	Ed *vs* Cor	0,99
	Ed *vs* Cau	0,0007
	Cap *vs* Cor	0,0119
	Cap *vs* Cau	0,0001
	Cor *vs* Cau	0,0001

Supplementary Figure S5. Immunolocalization of GIPC1 in the epididymal epithelium of control mice (*sv*/+) and Snell's waltzer mutants (*sv*/*sv*). The following panels show: caput (including the common efferent duct and initial segment), corpus, and cauda. GIPC1 is stained in *red*, F-actin is stained in *green*, and chromatin is stained in *blue*. Scale bar 50 µm. Graphs represent differences in fluorescence levels in individual segments (*Ed*, efferent duct; *Is*, initial segment; *Cap*, caput; *Cor*, corpus; *Cau*, cauda) in control and mutant mice. Error bars represent standard deviation, and statistical significance is indicated as $*p \leq 0.05$, $***p \leq 0.001$, and $****p \leq 0.0001$. Exact p-values are summarized in the table.

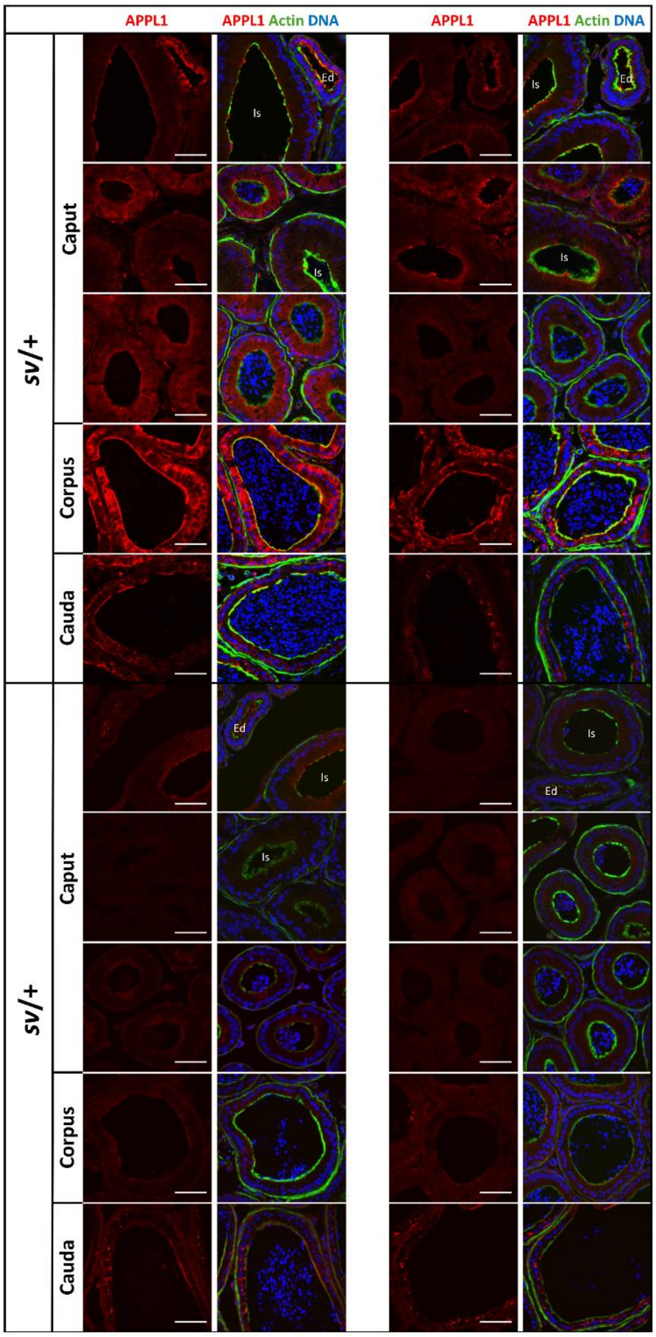

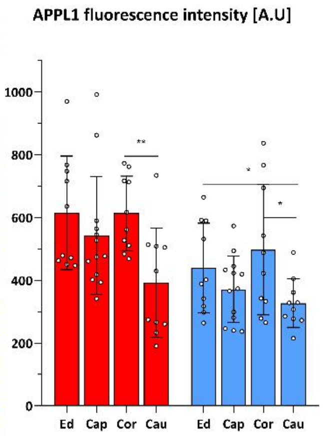

Group		p-values
		APPL1
sv/+	Ed *vs* Cap	0,4707
	Ed *vs* Cor	0,4002
	Ed *vs* Cau	0,0653
	Cap *vs* Cor	0,1151
	Cap *vs* Cau	0,0769
	Cor *vs* Cau	0,0045
sv/sv	Ed *vs* Cap	0,2292
	Ed *vs* Cor	0,4767
	Ed *vs* Cau	0,0485
	Cap *vs* Cor	0,1061
	Cap *vs* Cau	0,2578
	Cor *vs* Cau	0,0334

Supplementary Figure S6. Immunolocalization of APPL1 in the epididymal epithelium of control mice (*sv*/+) and Snell's waltzer mutants (*sv*/*sv*). The following panels show: caput (including the common efferent duct and initial segment), corpus, and cauda. APPL1 is stained in *red*, F-actin is stained in *green*, and chromatin is stained in *blue*. Scale bar 50 µm. Graphs represent differences in fluorescence levels in individual segments (*Ed*, efferent duct; *Is*, initial segment; *Cap*, caput; *Cor*, corpus; *Cau*, cauda) in control and mutant mice. Error bars represent standard deviation, and statistical significance is indicated as $*p \leq 0.05$, $**p \leq 0.01$. Exact p-values are summarized in the table.

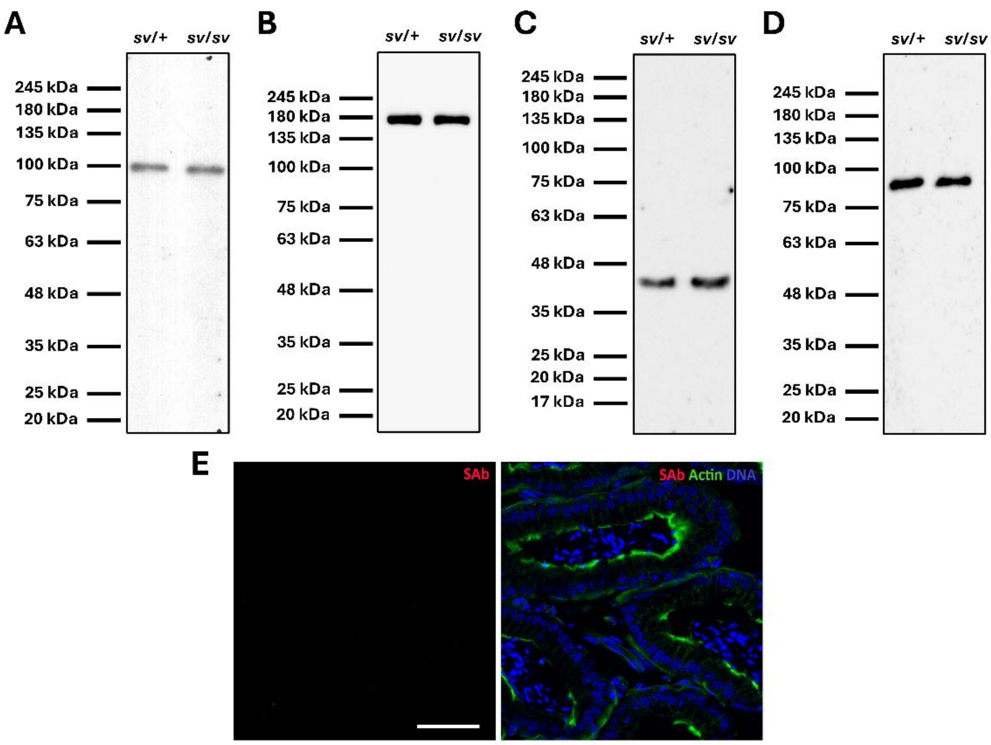

Supplementary Figure S7. Specificity of the primary antibodies by immunoblotting (**A-D**) and the negative control of immunofluorescence labeling (**E**). Immunoblotting of crude protein extracts from mouse epididymis (dissected from control *sv/*+ and MYO6 mutants *sv/sv*) with anti-Dab2 (**A**), anti-Clathrin (**B**), anti-GIPC1 (**C**), and anti-APPL1 (**D**) PAbs. The protein marker (Protein Marker VI, 10 – 245 kDa, pre-stained, AppliChem) is indicated on the left of each blot. The representative negative control with omitting the primary antibodies shows no signal for the secondary antibody (Sab, *red* fluorescence) in *sv*/+ epididymis (**E**). F-actin (*green*), nuclei (*blue*). Scale bar of 50 µm.

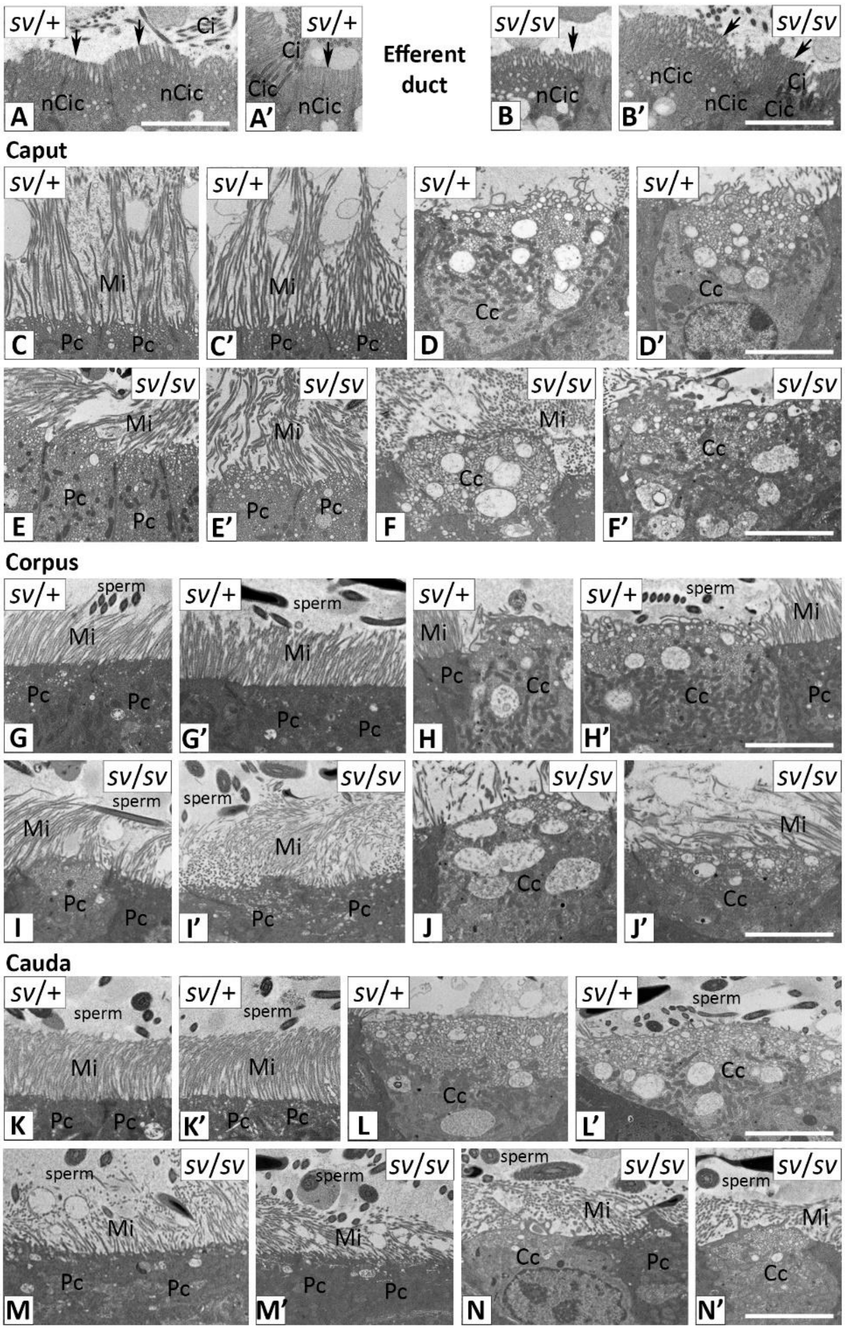

Supplementary Figure S8. Transmission electron microscopy of the apical epithelial zone of the common efferent duct and the epididymis in control (*sv*/+) and MYO6 mutant (*sv*/*sv*) male mice. Images **A**, **A'** (control) and **B**, **B'** (MYO6 mutant) show the ultrastructure of the apical epithelial zone of the efferent duct; *arrows* point to properly formed brush border structure in the nonciliated cells. Images **C**, **C'**, **G**, **G'**, **K**, **K'** (control) and **E**, **E'**, **I**, **I'**, **M**, **M'** (MYO6 mutant) show the ultrastructure of the apical zone of microvilli-containing principal cells, whereas images **D**, **D'**, **H**, **H'**, **L**, **L'** (control) and **F**, **F'**, **J**, **J'**, **N**, **N'** (MYO6 mutant) show the ultrastructure of clear cells in the following epididymal segments: caput, corpus, and cauda. *Cc*, clear cells; *Ci*, cilia; *Cic*, ciliated cell; *Mi*, microvilli; *nCiC*, nonciliated cells; *Pc*, principal cells. Scale bar 5 μm.
