# Supplementary material for: Myosin VI is involved in the structural organization and molecular composition of epididymal epithelial cells in mouse
Source: Biol Reprod. 2026 Feb 2;114(5):1747–64. doi: 10.1093/biolre/ioag031 (PMC13175989; doi:10.1093/biolre/ioag031)
Supplement: Supplementary_Tables_S1-S4_ioag031 [file supplementary_tables_s1-s4_ioag031.pdf]

Supplementary Table S1. Primary polyclonal antibodies (PAbs) used for immunofluorescence (IF), immunoblotting (IB) and immunoprecipitation (IP)

Target	Supplier	PAb Cat. No	RRID	Dilution		
				IF	IB	IP
MYO6	Proteintech	26778-1-AP	AB_2880631	1:200	1:2000	1:1500
Dab2		10109-2-AP	AB_2089700		1:1000	1:1000
Clathrin		26523-1-AP	AB_2880542		1:5000	x
GIPC1		14822-1-AP	AB_2263269		1:1000	1:1000
APPL1		12639-1-AP	AB_2289669		1:2000	x

Supplementary Table S2. List of primers used in RT-PCR experiments

Nested PCR	Forward primers	Reverse primers
**First PCR**	5′-GATGAGGCACAGGGTGAC-3′	5′-TTGTTCTGAGGGTCTTTGTA-3′
**Second PCR**	5′-ATGAGGCACAGGGTGACAT-3′	5′-TTCTGAGGGTCTTTGTACTGGT-3′

Supplementary Table S3. Summary of p-values for statistical analyses comparing differences in testicular and epididymal weights and body weight ratios between control and mutant mice

Weight [mg]		p-values	Weight ratio [mg/g]		p-values
**Testes**	***sv/+ vs sv/sv***	0,007	**Testes/Body**	***sv/+ vs sv/sv***	0,0083
**Epididymides**		0,0185	**Epididymides/Body**		0,0001

Supplementary Table S4. Summary of p-values for statistical analyses comparing differences in immunofluorescence intensity of selected proteins in the efferent duct and epididymal segments between control and mutant mice

Group		p-values			
		Dab2	CLTC	GIPC1	APPL1
**Ed**	***sv/+ vs sv/sv***	0,0001	0,0440	0,297	0,0288
**Cap**		0,0908	0,0001	0,009	0,0102
**Cor**		0,0524	0,0132	0,0012	0,1453
**Cau**		0,0753	0,007	0,8443	0,3038
